# Supplementary figures and images for: Local chromatin context regulates the genetic requirements of the heterochromatin spreading reaction
Source: PLoS Genet. 2022 May 18;18(5):e1010201. doi: 10.1371/journal.pgen.1010201 (PMC9154106; doi:10.1371/journal.pgen.1010201)

# S1 Fig

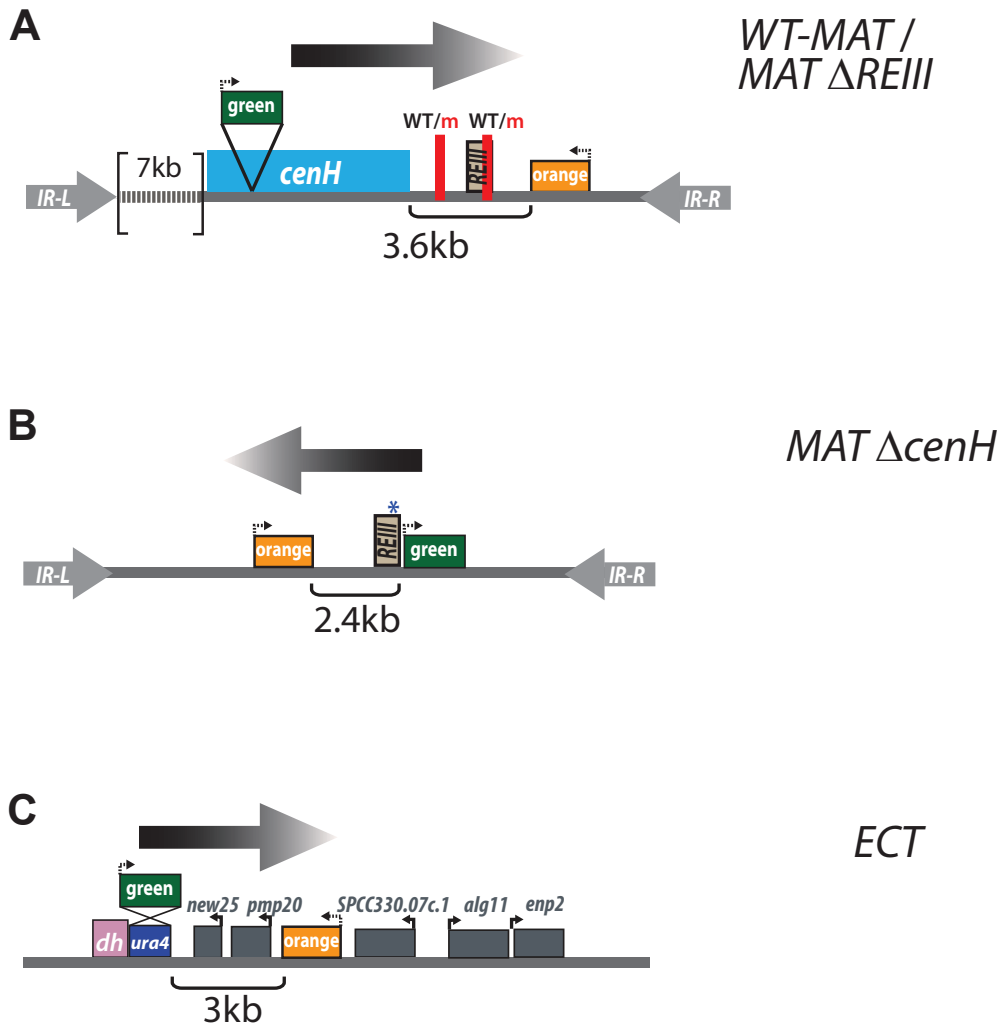

Supplement: S1 Fig — To-scale diagrams of the heterochromatin spreading sensors (without the euchromatically placed “red” reporter) in the 4 chromatin contexts used for the spreading screen (as in Greenstein et al 2018). The direction in which spreading is analyzed (“green” to “orange”) is indicated per chromatin context. A. WT MAT and MAT ΔREIII. These two contexts are similar, except that MAT ΔREIII contains two short 7bp deletions of the two Atf1/Pcr1 DNA binding sites near REIII, inactivating it. The first binding site is not included in REIII, per the definitions of [98] and [23]. B. MAT ΔcenH. C. ECT. In Greenstein et al 2018, the distance between “green” and “orange” was varied for B. and C. contexts, without changes to the qualitative behavior of spreading. (PDF) [file pgen.1010201.s001.pdf]

S2 Fig

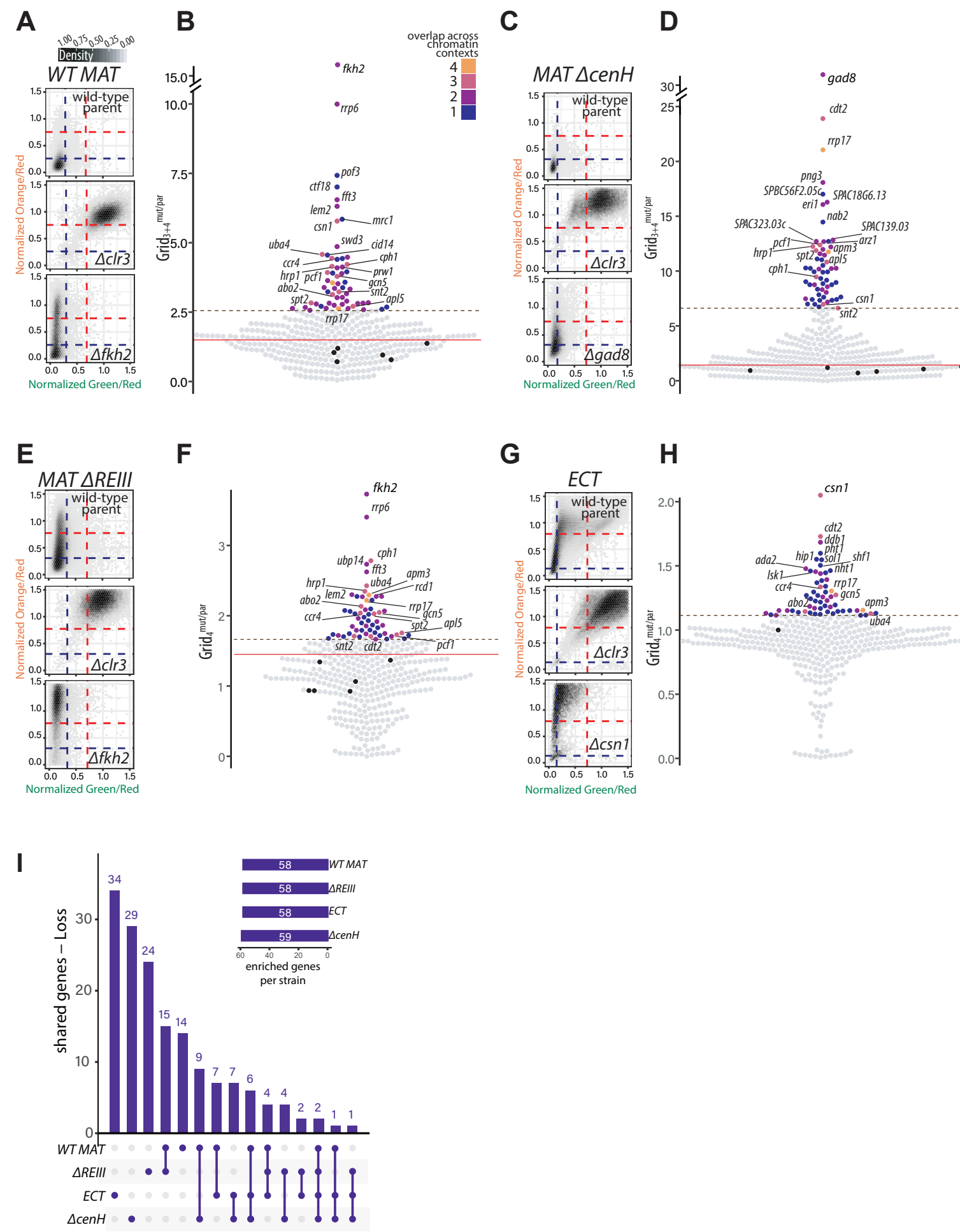

Supplement: S2 Fig — A. WT-MAT 2D-density hexbin plots of the wild-type parent, a strong heterochromatin loss hit (Δclr3), and the top loss of spreading hit (Δfkh2) in this chromatin context. Dashed blue lines indicate the values for repressed fluorescence state and dashed red lines indicate values for fully expressed fluorescence state. B. Beeswarm plots of Grid3+4mut/par for WT MAT loss of spreading hits. The top 10 hits are all annotated, and below those hits, mutants that show overlap with at least 3 other chromatin contexts are additionally annotated. Red line, 2SD above the Grid3+4mut/par of the wild-type parent isolates (black dots); dashed brown line, the 85th percentile; Dot color, number of chromatin contexts with loss of spreading phenotype over the cutoff. C.-D. Data for the WT ΔcenH strain were analyzed and displayed as in E. and F. This mutation allows examination of only the REIII nucleation site at the MAT locus. E.-F. Data for the WT ΔREIII strain were analyzed and displayed as in E. and F expect that Grid4mut/par was used as the metric. G.-H. Data for the ECT strain were analyzed and displayed as in E. and F.I. Upset plots indicating the frequency of “loss of spreading” gene hits appearing in one or multiple chromatin contexts. For each bar, the chromatin context(s) with shared phenotypes for the underlying gene hits is indicated below the plot. The inset indicates the total number gene hits for loss of spreading in each chromatin context. “Shared genes”: number of genes that appear as “loss of spreading” hits across the number of indicated chromatin contexts. (PDF) [file pgen.1010201.s002.pdf]

# S3 Fig

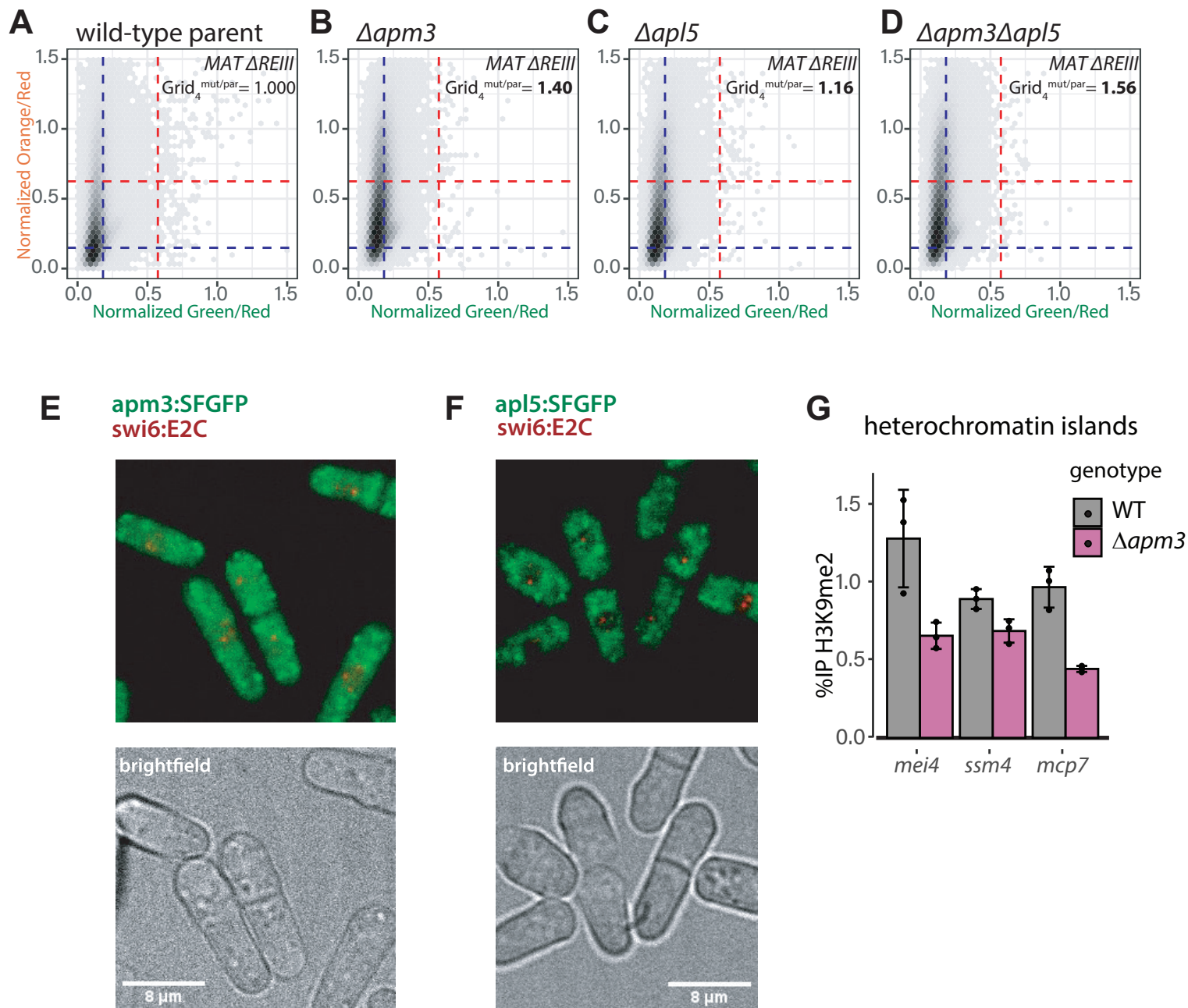

Supplement: S3 Fig — A.-D. 2D density hexbin plots of de novo generated Δapm3 (B.), Δapl5 (C.), and Δapm3Δapl5 double mutant (D.) compared to the wild-type MAT ΔREIII parent (A.). The Fold change of Grid4mut/par is indicated in the plot. At least 3 independent isolates of each genotype are combined in each plot. E. Apm3:SFGFP is distributed in the cytosol and nucleus. Apm3:SFGFP was expressed from its native locus and co-expressed with Swi6:E2C. Swi6:E2C labels nuclear heterochromatin. Z-projection overlays of the Apm3:SFGFP and Swi6:E2C on top, and a brightfield image on the bottom. F. Apl5:SFGFP is largely nuclear excluded. Apl5:SFGFP was expressed from its native locus and co-expressed with Swi6:E2C. Swi6:E2C labels nuclear heterochromatin. Z-projection overlays of the Apl5:SFGFP and Swi6:E2C on top, and a brightfield image on the bottom. G. Δapm3 exhibits a mild defect in H3K9me2 accumulation at heterochromatin islands. H3K9me2 ChIP-qPCR in wild-type parent MAT ΔREIII or Δapm3 mutant. Error bars represent 1SD of three replicates. (PDF) [file pgen.1010201.s003.pdf]

S4 Fig

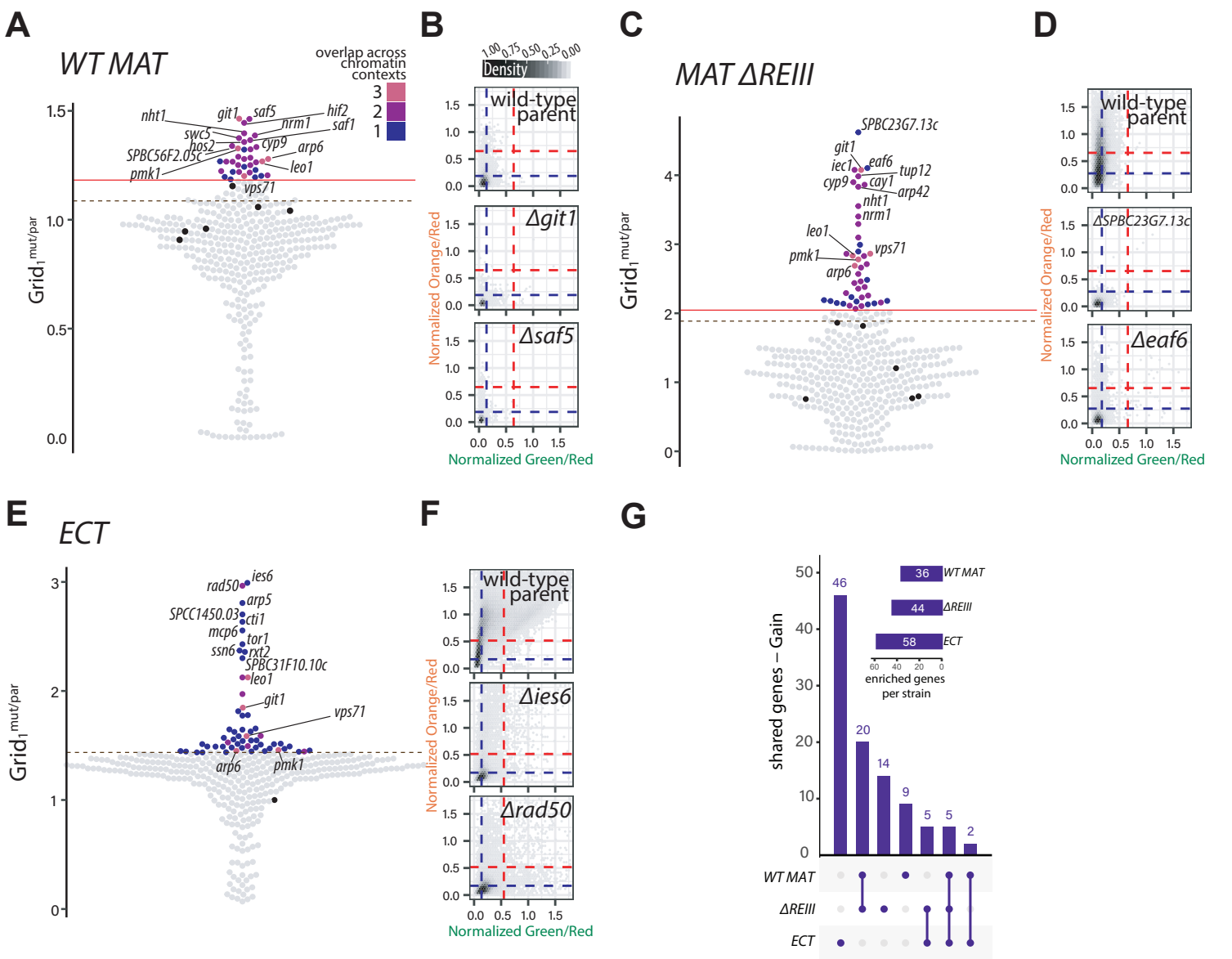

Supplement: S4 Fig — A. Beeswarm plots of Grid1mut/par for WT MAT gain of nucleation-distal silencing hits. The top 10 hits are all annotated, and below those hits, mutants that show overlap with 2 other chromatin contexts are additionally annotated. Red line, 2SD above the Grid1mut/par of wild-type parent isolates (black dots); dashed brown line, the 85th percentile; Dot color, number of chromatin contexts with loss of spreading phenotype over the cutoff. B. WT MAT 2D-density hexbin plots of the wild-type parent, and the two top gain of nucleation-distal silencing hits of this chromatin context. Dashed blue lines indicate the values for repressed fluorescence state and dashed red lines indicate values for fully expressed fluorescence state. C.-D. As in A., B. but for MAT ΔREIII. E.-F. As in A., B. but for ECT. H. Upset plots indicating the frequency of gain of nucleation-distal silencing gene hits that appear in the chromatin the three in contexts as in Fig 2B. For each bar, the chromatin context(s) with shared phenotypes for the underlying gene hits is indicated below the plot. The inset indicates the total number gene hits in each chromatin context of the same phenotype. (PDF) [file pgen.1010201.s004.pdf]

## S5 Fig

Gain of distal silencing mutants:

Loss of distal silencing mutants:

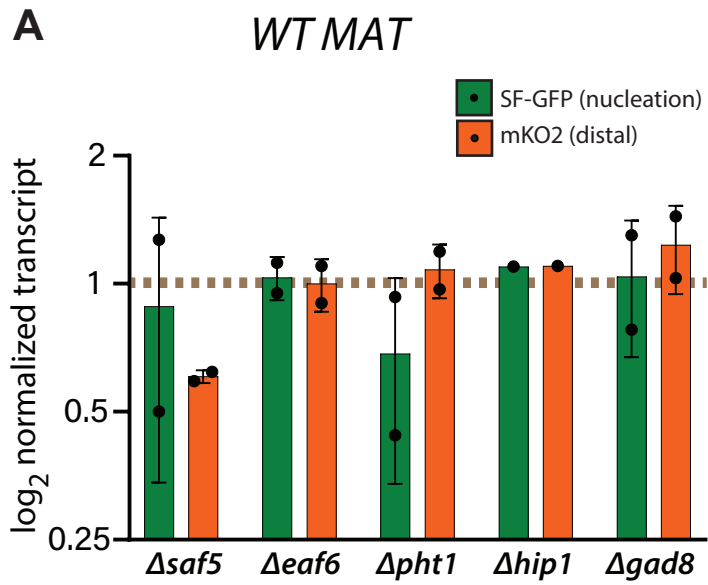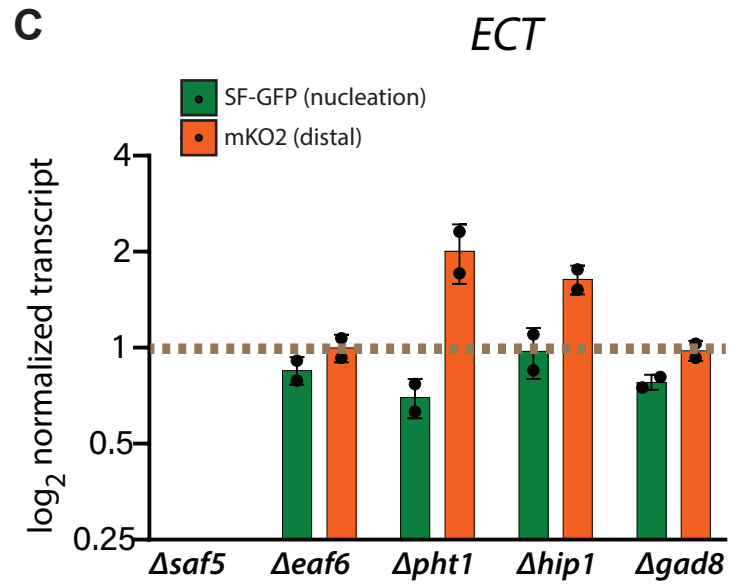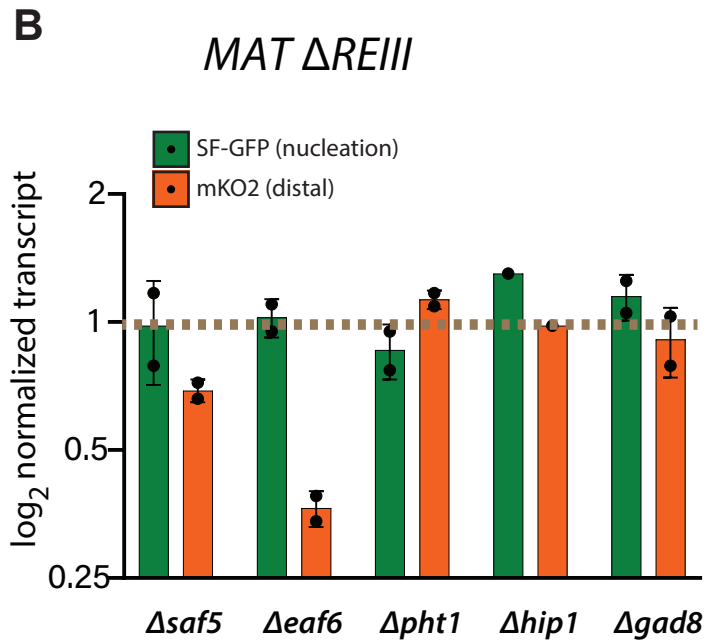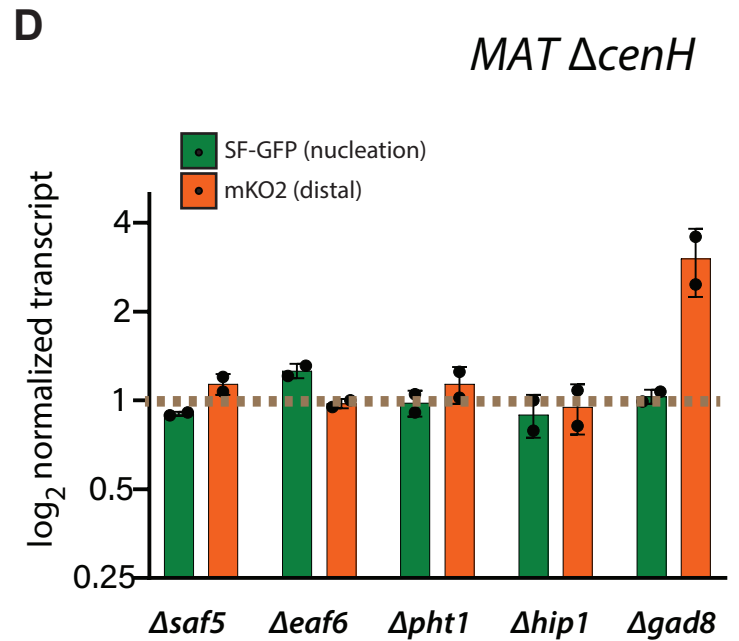

Supplement: S5 Fig — 5 moderate- to strong hits in the loss and gain of distal silencing category that are partially or fully chromatin context-specific were selected for validations: saf5 (gain of silencing in WT MAT, and moderately in MAT ΔREIII), eaf6 (gain of silencing only in MAT ΔREIII), pht1 and hip1 (loss of silencing only in ECT), and gad8 (strong loss of silencing in MAT ΔcenH and mildly in ECT). RT-qPCRs for SF-GFP (“green”-nucleation) and mKO2 (“orange”, distal) transcripts normalized to the act1 transcript and scaled to the wild-type (dashed brown line, = 1) are shown for examples of: Gain of distal silencing; A. WT-MAT, B. MAT ΔREIII. Loss of distal silencing; C. ECT, D. MAT ΔcenH. Error bars indicate 1SD of 2 biological replicates generated independently from the screen. Dotted lines represent wild-type control. Note we could not recover Δsaf5 mutants in ECT. (PDF) [file pgen.1010201.s005.pdf]

S6 Fig

A

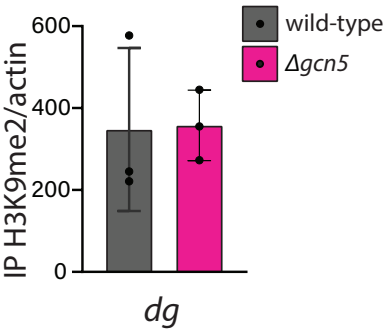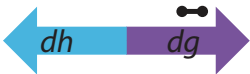

B

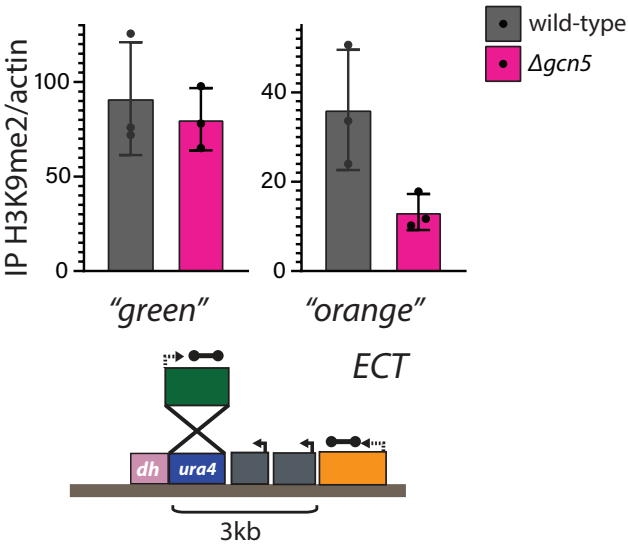

Supplement: S6 Fig — act1-normalized ChIP-qPCR for H3K9me2 in ECT wild-type parent or the de novo generated Δgcn5 mutant at A. the pericentomeric dg element, and B. the heterochromatin spreading sensor at the ura4 locus in ECT. Dumbbells indicate qPCR amplicons. Error bars indicate 1SD of 3 biological replicates. (PDF) [file pgen.1010201.s006.pdf]

S7 Fig

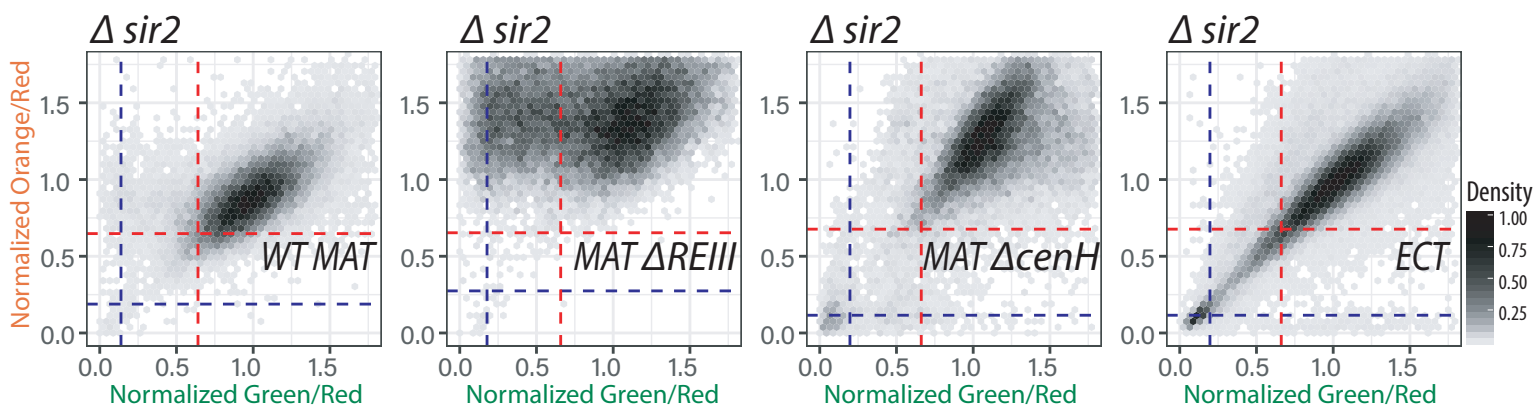

Supplement: S7 Fig — 2D density hexbin plots of Δsir2 mutants in each chromatin context from the screen. Mutation in sir2 causes a loss of silencing phenotype in all examined chromatin context. (PDF) [file pgen.1010201.s007.pdf]

S8 Fig

*MAT ΔREIII* - Clr6 Subcomplex Components

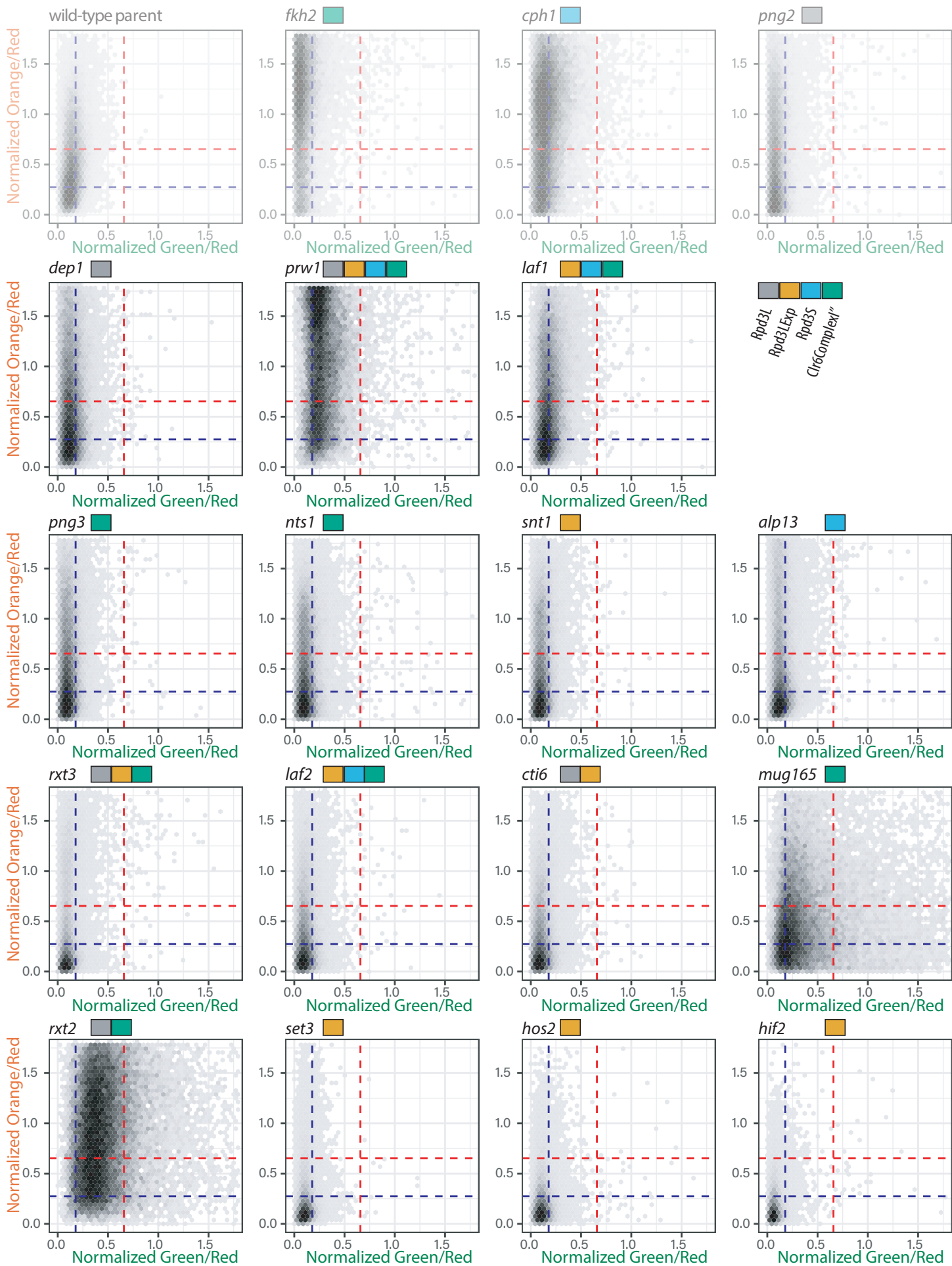

Supplement: S8 Fig — 2D density hexbin plots of all Clr6 complexes gene mutants from the screen, corresponding to Fig 4E in MAT ΔREIII context. The mutants are arranged in descending order of Grid3+4mut/par; in MAT ΔREIII only Δfkh2, Δcph1, Δpng2, Δdep1, Δprw1 and Δlaf1 were identified as loss of spreading phenotype. Original MAT ΔREIII wild type parent and mutants shown in Figs 1 and 4E are shown here again (with transparency) for comparison. GO complex annotations are indicated next to each mutant by colored boxes. (PDF) [file pgen.1010201.s008.pdf]

# S9 Fig

**A**

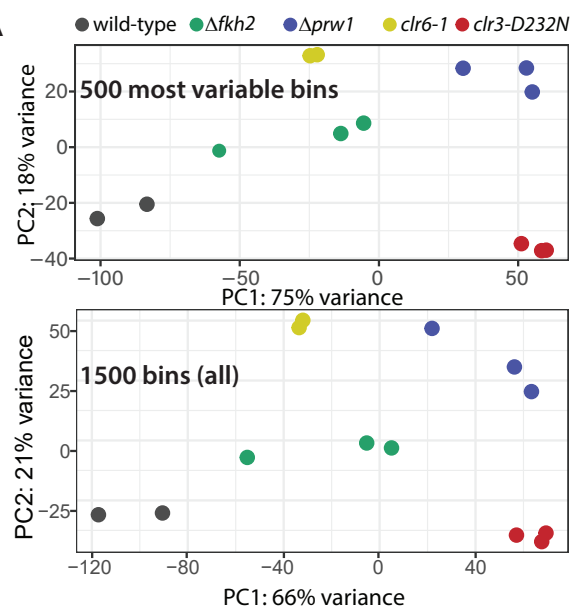

**B**

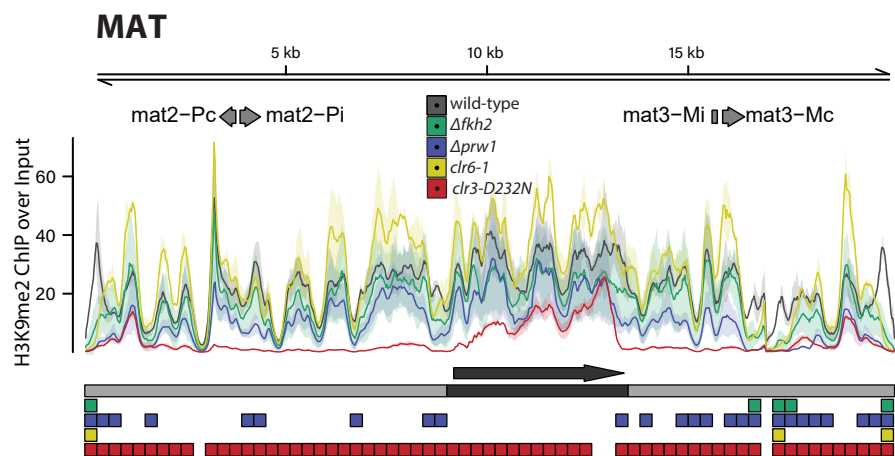

**C**

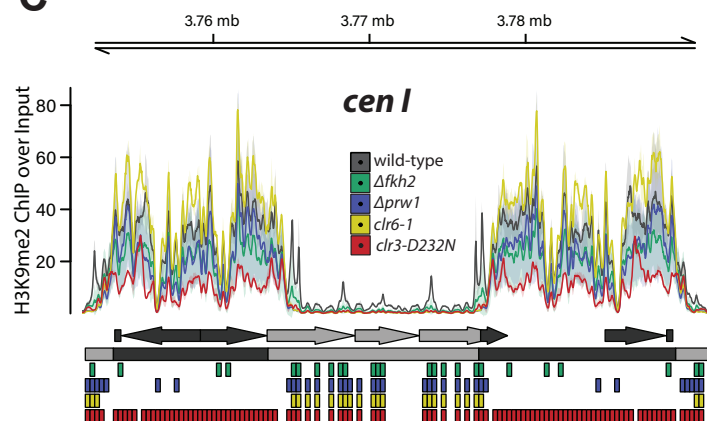

**D**

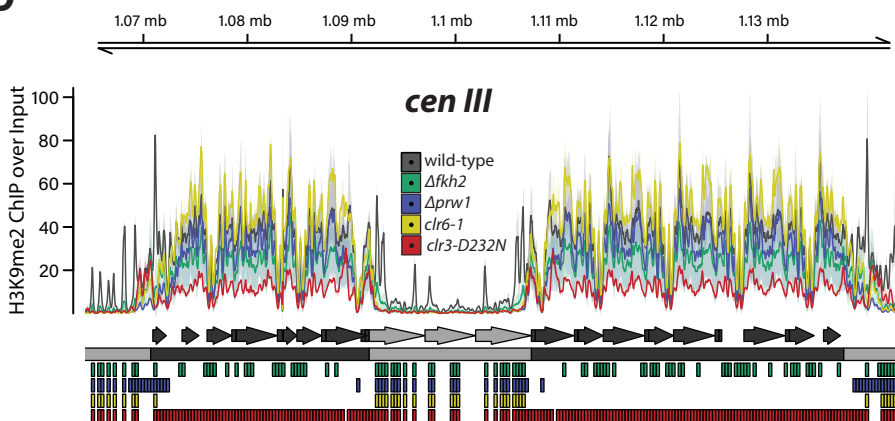

**E**

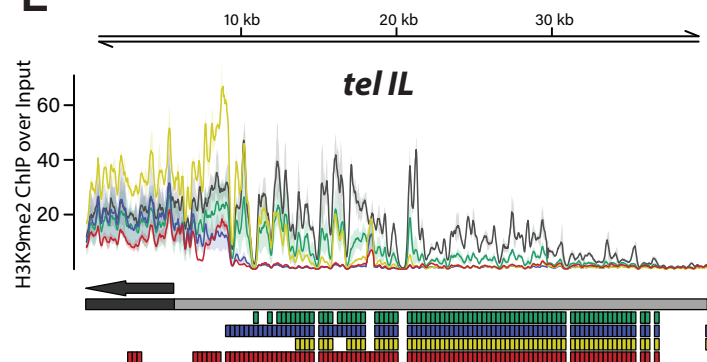

**F**

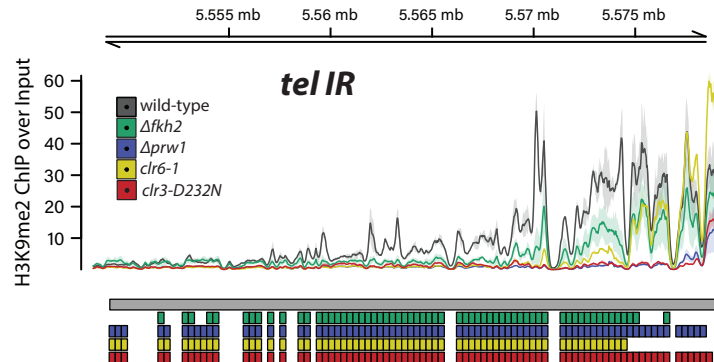

Supplement: S9 Fig — A. Principal Component Analysis was performed on the normalized counts in the top 500 most variable bins (analogous to RNA-Seq analysis, TOP) or all 1500bp bins (BOTTOM) passing a threshold for global enrichment of H3K9me2 signal (see Materials and Methods). The first two principal component values are plotted for each sample with genotypes as defined in the legend. B.-F. Signal tracks plots for the MAT locus and indicated centromeres and telomeres as in the main text. No nucleator sequences are present on subtelomere IR so the first annotation row below the signal tracks is empty. (PDF) [file pgen.1010201.s009.pdf]

**S10 Fig****A**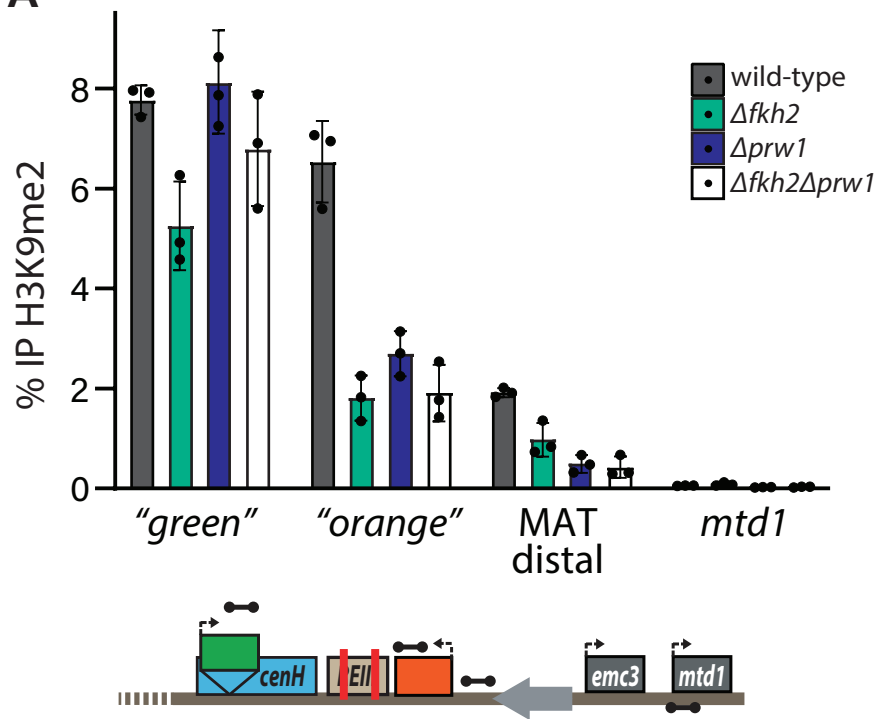**B**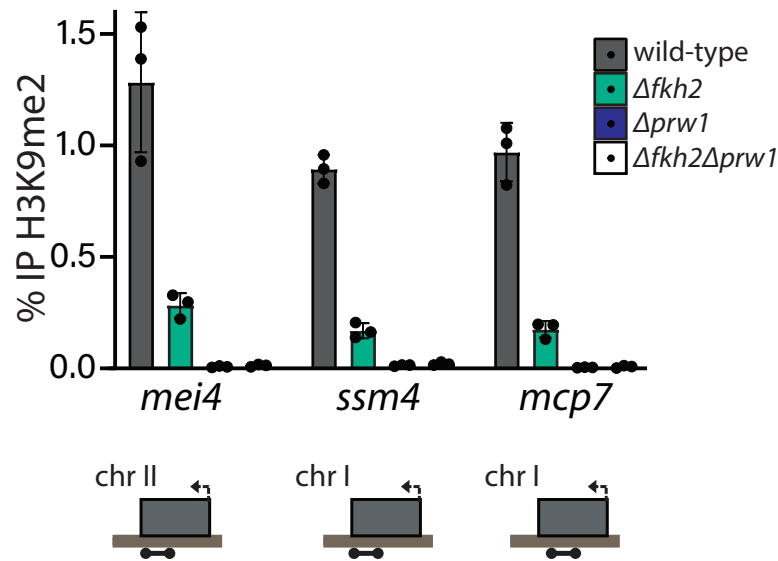**C**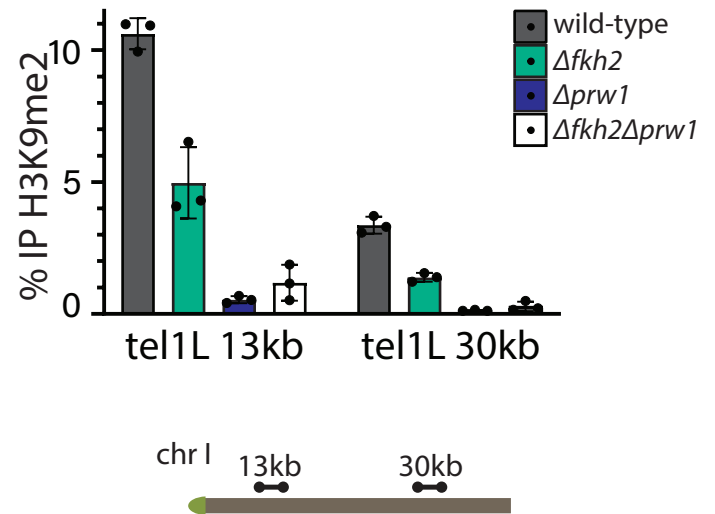

Supplement: S10 Fig — A. H3K9me2 ChIP-qPCR at the MAT locus in wild-type MAT ΔREIII, Δfkh2, Δprw1, and the Δfkh2Δprw1 double mutant. B. As in A., at indicated heterochromatin islands. C. As in A., at tel IL. Error bars represent 1SD of 3 biological replicates. (PDF) [file pgen.1010201.s010.pdf]

S11 Fig

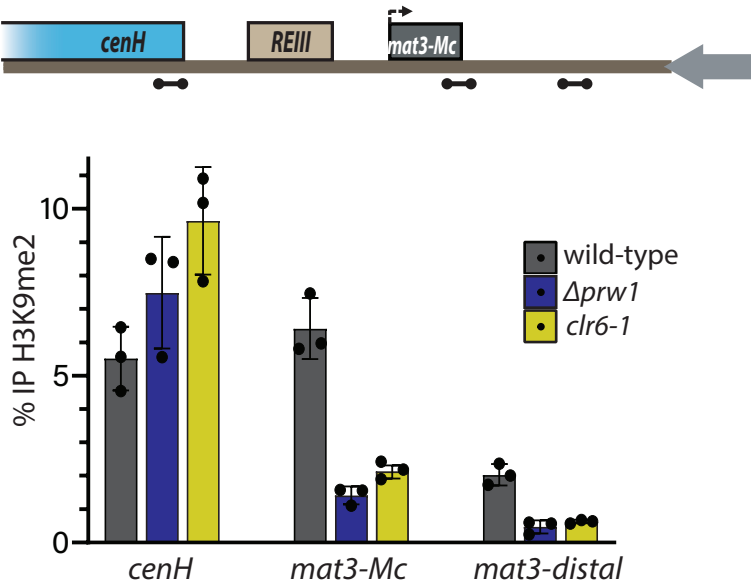

Supplement: S11 Fig — H3K9me2 ChIP-qPCR at the MAT locus in a wild-type MAT locus (no heterochromatin spreading reporters, see diagram), Δprw1, and clr6-1, at indicated amplicons (dumbbells). Error bars represent 1SD of 3 biological replicates. (PDF) [file pgen.1010201.s011.pdf]

S12 Fig

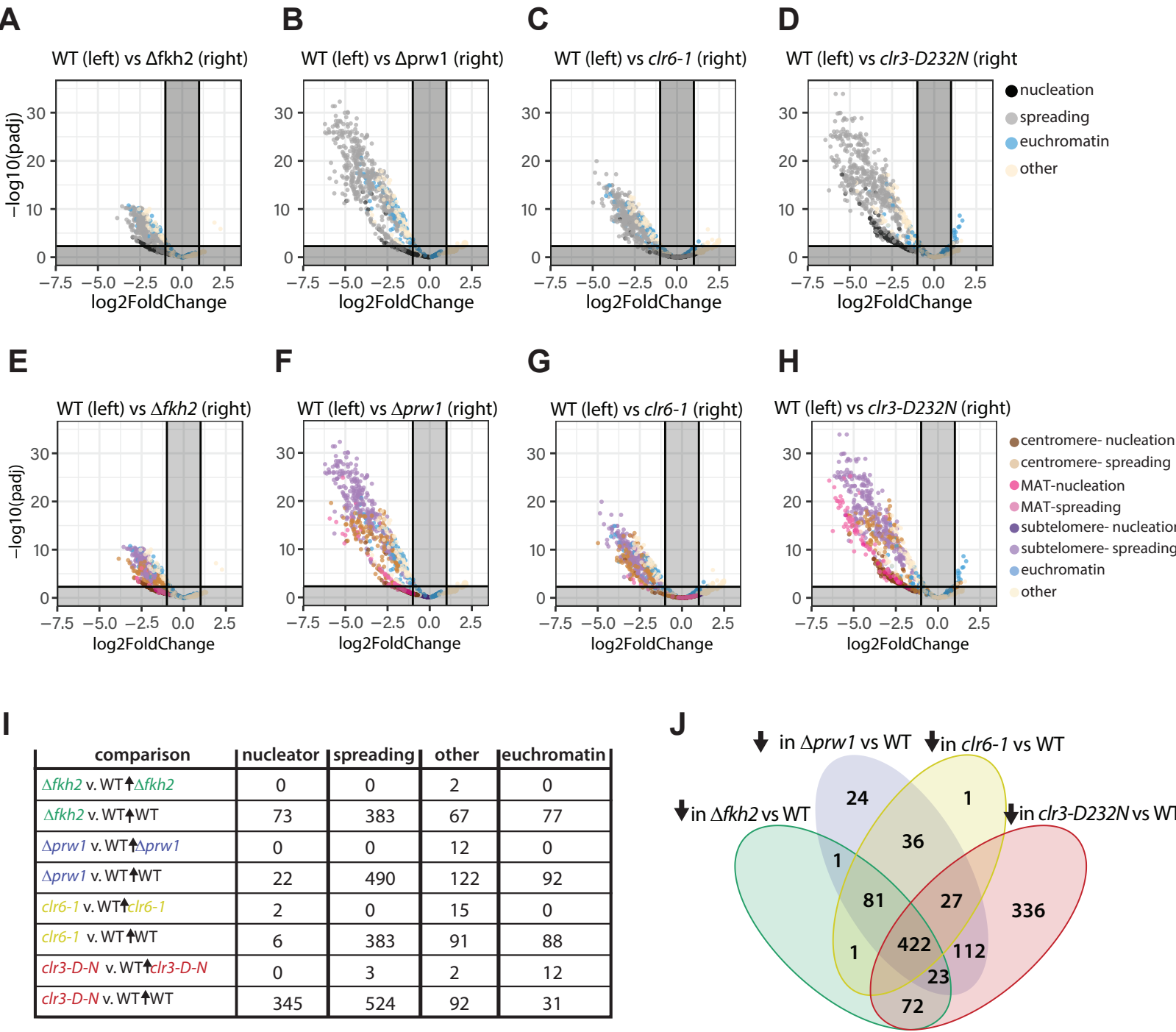

Supplement: S12 Fig — A.-D. Volcano plots representing -log10(adjusted p-value) vs log2FoldChange values for mutants (I., Δfkh2; J., Δprw1; K, clr6-1; L., clr3-D232N) over WT. P-values were corrected for multiple testing with the Benjamini-Hochberg procedure. Cutoff values for adjusted p-value < 0.005 and absolute value Log2FoldChange > 1 are annotated on the plot. Dots represent individual 300bp windows tested for differential enrichment. Dots are colored by their annotation to nucleation or spreading zones, presence within a previously identified euchromatin embedded H3K9me2 heterochromatin region (“island”, “HOOD”, or “region”), or regions outside these categories (other). E.-H. Volcano plots were generated as in A-D. Dots are colored by their annotation to nucleation or spreading zones broken down by heterochromatin location (pericentromere, subtelomere, MAT) or presence within a previously identified euchromatin embedded H3K9me2 heterochromatin region. I. The number of regions called as significant in each direction for each of the pairwise comparisons is tabulated per each category of genomic feature. J. The overlap of regions identified as significantly reduced in H3K9me2 signal in each mutant vs WT is compared in a Venn Diagram. (PDF) [file pgen.1010201.s012.pdf]

**S13 Fig**

**A**

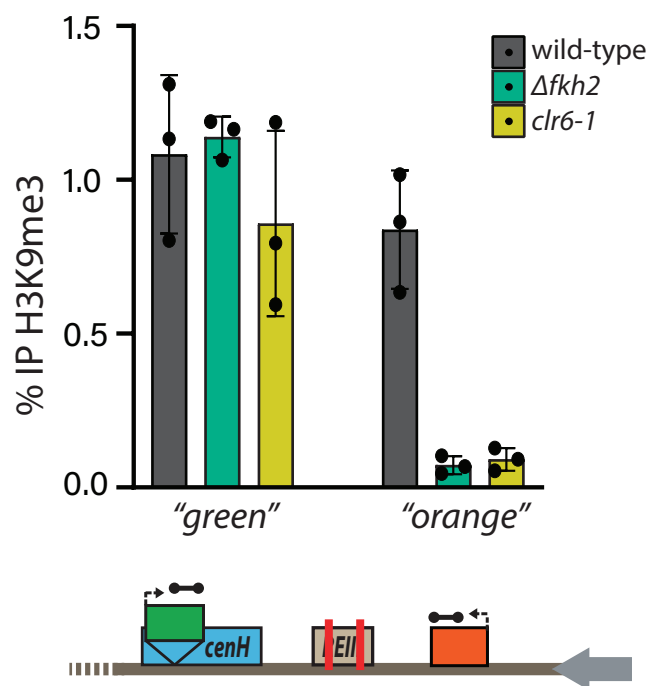

**B**

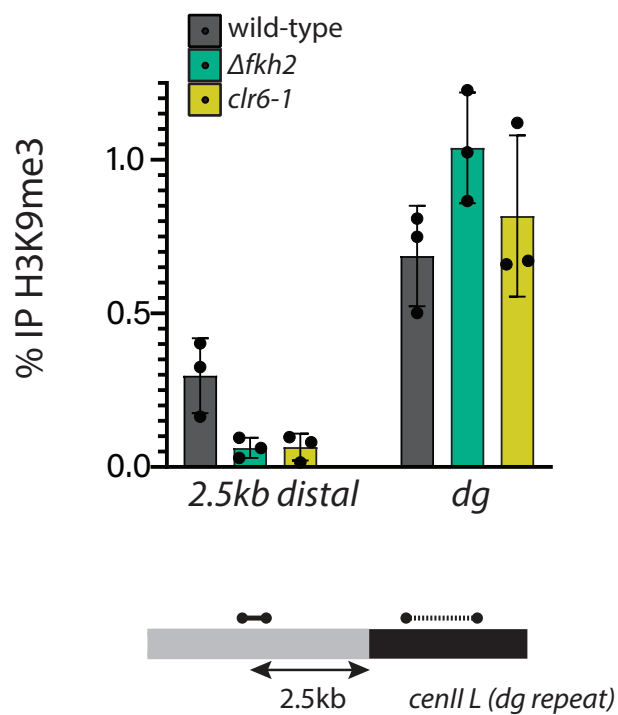

**C**

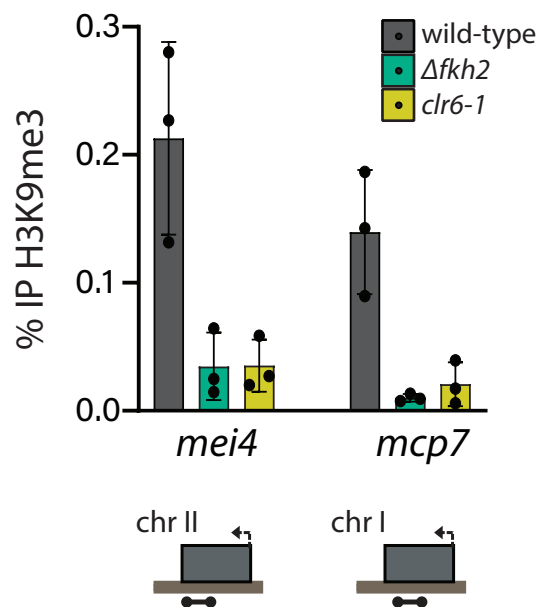

**D**

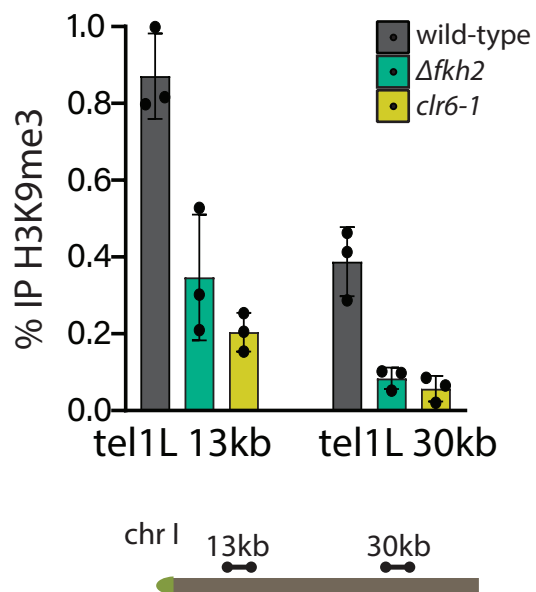

Supplement: S13 Fig — A. H3K9me3 ChIP-qPCR at the MAT locus in wild-type MAT ΔREIII, Δfkh2, and clr6-1. B. As in A., at dg repeats, which are at the distal end of the left of the pericentromere at cen II and an amplicon 2.5kb beyond the last annotated nucleating feature at cen II left. C. As in A., but at heterochromatin islands mei4 and mcp7. D. As in A., but at tel 1L. Error bars represent 1SD of 3 biological replicates. (PDF) [file pgen.1010201.s013.pdf]

S14 Fig

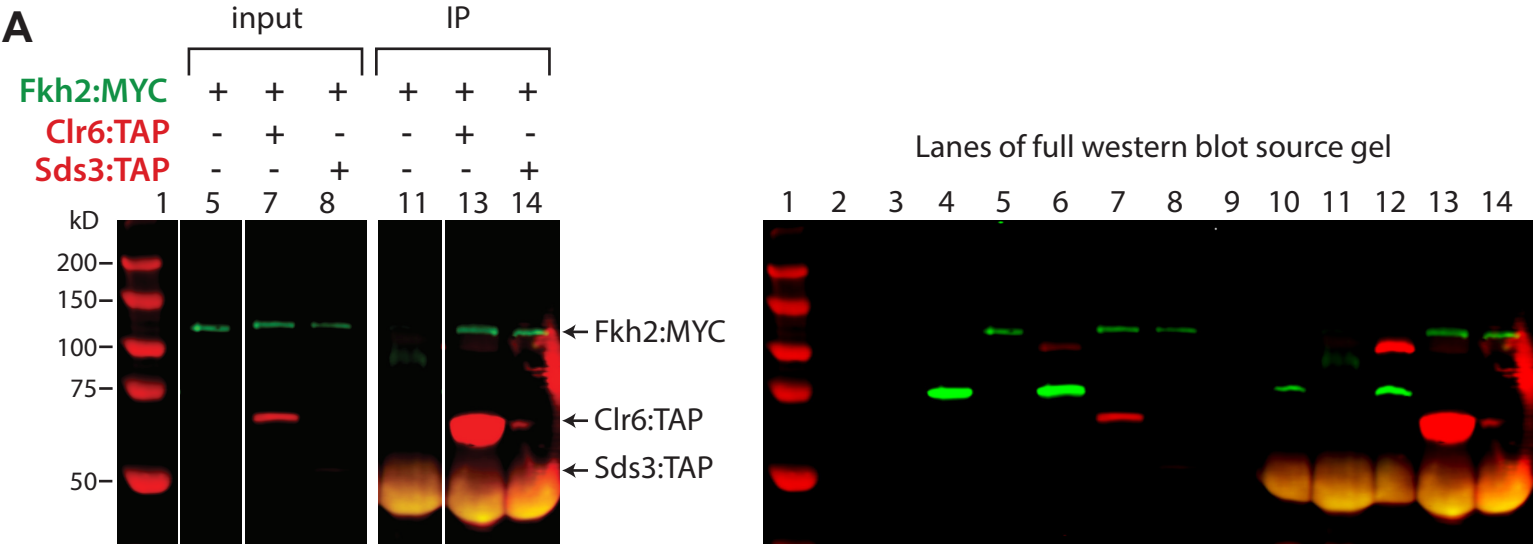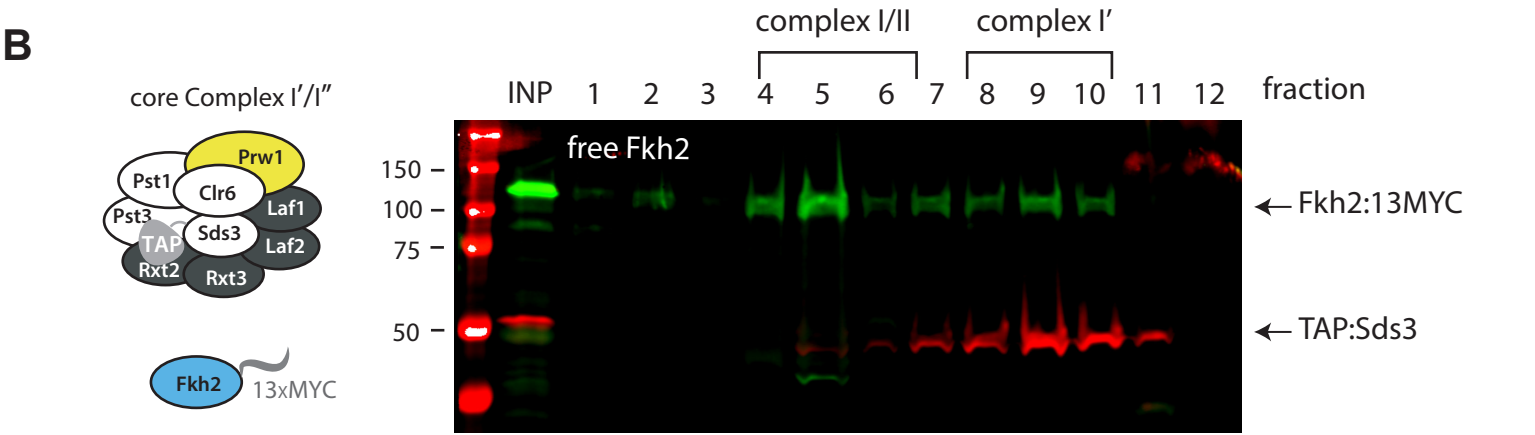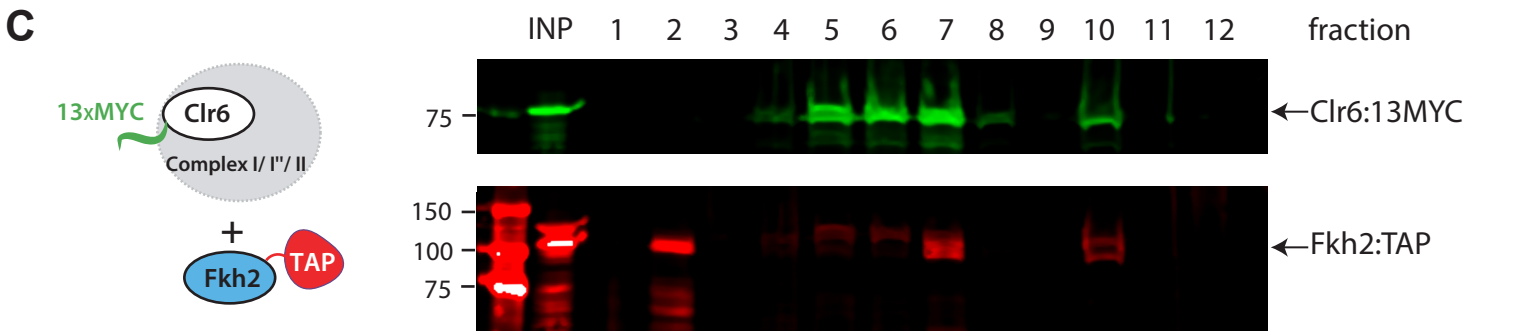

Supplement: S14 Fig — A. Co-Immunoprecipitation experiment with baits Clr6-TAP or Sds3-TAP and prey Fkh2-MYC. LEFT: Western blot against indicated proteins for the Co-IP experiment. RIGHT shows the entire western blot, including lanes unrelated to the co-IP experiment (2–4,5,10,12). B. Sucrose density gradient for whole cell extracts of cells containing Sds3-TAP, a signature of complex I/I″, and Fkh2:MYC. Gradient and Western as in Fig 6. C. Single channel Western blots of Fig 6A. (PDF) [file pgen.1010201.s014.pdf]

S15 Fig

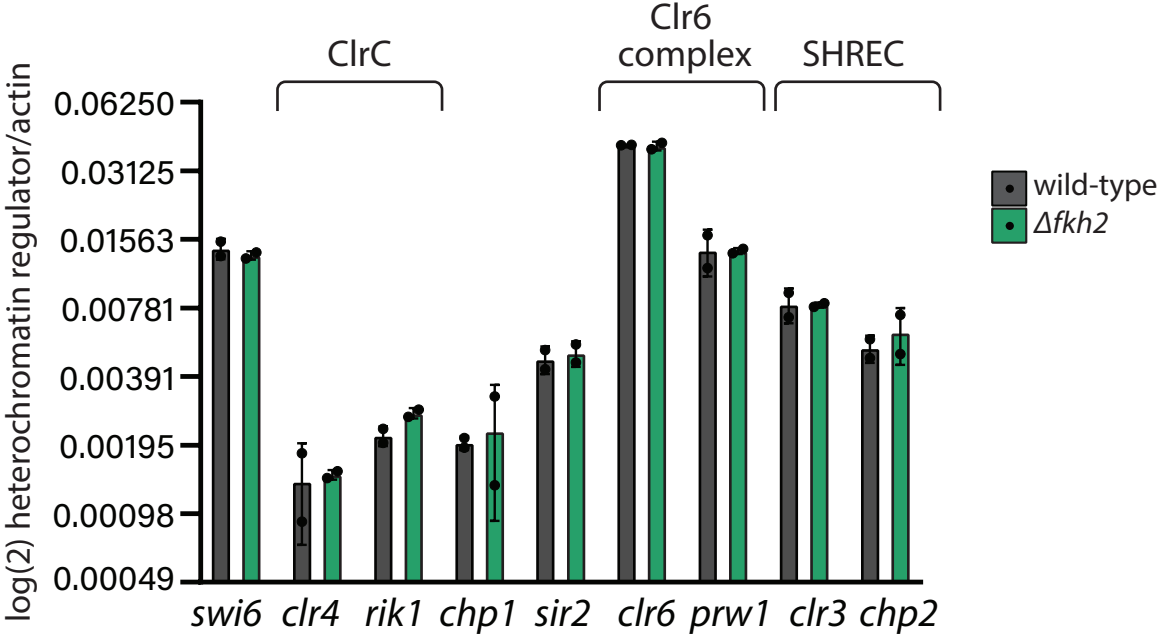

Supplement: S15 Fig — RT-qPCR of indicated core heterochromatin regulators, including representatives of ClrC, Clr6, and SHREC in MAT ΔREIII wild-type or Δfkh2 cells. Heterochromatin regulator transcripts are normalized to the act1 transcript and shown on a log2 scale, given the wide distribution of transcript abundance between indicated regulators. Error bars indicate 1SD of 2 biological replicates. (PDF) [file pgen.1010201.s015.pdf]
