# Supplementary material for: Local chromatin context regulates the genetic requirements of the heterochromatin spreading reaction
Source: PLoS Genet. 2022 May 18;18(5):e1010201. doi: 10.1371/journal.pgen.1010201 (PMC9154106; doi:10.1371/journal.pgen.1010201)
Supplement: S1 Table — List of gene deletion strains used for genetic screens in this study. (PDF) [file pgen.1010201.s016.pdf]

S1 Table: Nuclear function gene deletion library

	Systematic ID	Symbol	Description
1	SPAC1002.05c	jmj2	histone demethylase Jmj2
2	SPAC1006.03c	red1	RNA elimination defective protein Red1
3	SPAC1039.05c	klf1	transcription factor, zf-fungal binuclear cluster type Klf1
4	SPAC1071.02	mms19	CIA machinery protein Mms19
5	SPAC1071.06	arp9	SWI/SNF and RSC complex subunit Arp9
6	SPAC10F6.08c	nht1	Ino80 complex HMG box subunit Nht1
7	SPAC10F6.11c	atg17	autophagy associated protein kinase activator Atg17
8	SPAC1142.03c	swi2	Swi5 complex subunit Swi2
9	SPAC1142.08	fhl1	forkhead transcription factor Fhl1
10	SPAC11D3.07c	toe4	transcription factor, zf-fungal binuclear cluster type(predicted)
11	SPAC11D3.16c		Schizosaccharomyces specific protein
12	SPAC11E3.01c	swr1	SNF2 family ATP-dependent DNA helicase Swr1
13	SPAC11H11.01	sst6	ESCRT I complex subunit Vps23
14	SPAC11H11.05c	fta6	Mis6-Sim4 complex Fta6
15	SPAC12B10.10	nod1	medial cortical node Gef2-related protein protein Nod1
16	SPAC12G12.13c	cid14	TRAMP complex poly(A) polymerase subunit Cid14
17	SPAC139.03	toe2	transcription factor, zf-fungal binuclear cluster type (predicted)
18	SPAC139.06	hat1	histone acetyltransferase Hat1
19	SPAC1399.05c	toe1	transcription factor, zf-fungal binuclear cluster type
20	SPAC13A11.04c	ubp8	SAGA complex ubiquitin C-terminal hydrolase Ubp8
21	SPAC13D6.02c	byr3	translational activator, zf-CCHC type zinc finger protein (predicted)
22	SPAC13G6.01c	rad8	ubiquitin-protein ligase E3/ ATP-dependent DNA helicase Rad8
23	SPAC144.02	iec1	Ino80 complex subunit Iec1
24	SPAC144.05		DNA-dependent ATPase/ ubiquitin-protein ligase E3 (predicted)
25	SPAC144.06	apl5	AP-3 adaptor complex subunit Apl5 (predicted)
26	SPAC144.14	klp8	kinesin-like protein Klp8
27	SPAC14C4.06c	nab2	poly(A) binding protein Nab2 (predicted)
28	SPAC14C4.12c	laf1	Clr6 L associated factor 1 Laf1
29	SPAC14C4.13	rad17	RFC related checkpoint protein Rad17
30	SPAC1556.01c	rad50	DNA repair protein Rad50
31	SPAC15A10.11	ubr11	UBR ubiquitin-protein ligase E3 Ubr11
32	SPAC15A10.15	sgo2	inner centromere protein, shugoshin Sgo2
33	SPAC1610.01	saf5	splicing factor Saf5
34	SPAC1610.02c	mrpl1	mitochondrial ribosomal protein subunit L1 (predicted)
35	SPAC1687.05	pli1	SUMO E3 ligase Pli1
36	SPAC1687.09	irs4	autophagy/CVT pathway ENTH/VHS domain protein Irs4 (predicted)
37	SPAC16A10.03c		ubiquitin-protein ligase E3 involved in vesicle docking Pep5/Vps11-like (predicted)
38	SPAC16A10.07c	taz1	shelterin complex subunit Taz1
39	SPAC16C9.04c	mot2	CCR4-Not complex ubiquitin-protein ligase E3 subunit Mot2
40	SPAC16C9.05	cph1	Clr6 histone deacetylase associated PHD protein-1 Cph1
41	SPAC16E8.12c	png3	ING family homolog Png3 (predicted)
42	SPAC1751.01c	gti1	gluconate transmembrane transporter inducer Gti1
43	SPAC1782.05	ypa2	protein phosphatase type 2A regulator, PTPA family Ypa2
44	SPAC1782.08c	rex3	exonuclease Rex3 (predicted)
45	SPAC1782.09c	clp1	Cdc14-related protein phosphatase Clp1/Flp1
46	SPAC1783.05	hrp1	ATP-dependent DNA helicase Hrp1
47	SPAC17A2.12	rrp1	ATP-dependent DNA helicase/ ubiquitin-protein ligase E3 (predicted)
48	SPAC17G8.05	med20	mediator complex subunit Med20
49	SPAC17G8.07	yaf9	YEATS family histone acetyltransferase subunit Yaf9
50	SPAC17G8.09	shg1	Set1C complex subunit Shg1
51	SPAC17G8.10c	dma1	mitotic spindle checkpoint ubiquitin ligase Dma1
52	SPAC17G8.13c	mst2	histone acetyltransferase Mst2
53	SPAC17H9.10c	ddb1	Cul4-RING E3 adaptor Ddb1
54	SPAC17H9.19c	cdt2	WD repeat protein Cdt2
55	SPAC1805.14		Schizosaccharomyces specific protein
56	SPAC1805.15c	pub2	HECT-type ubiquitin-protein ligase E3 Pub2
57	SPAC1851.03	ckb1	CK2 family regulatory subunit Ckb1
58	SPAC18G6.02c	chp1	heterochromatin (HP1) family chromodomain protein Chp1
59	SPAC18G6.10	lem2	LEM domain nuclear inner membrane protein Heh1/Lem2
60	SPAC18G6.13		Schizosaccharomyces specific protein
61	SPAC1952.05	gcn5	SAGA complex histone acetyltransferase catalytic subunit Gcn5
62	SPAC19A8.10	rfp1	SUMO-targeted ubiquitin-protein ligase subunit Rfp1
63	SPAC19D5.06c	din1	RNA pyrophosphohydrolase Din1
64	SPAC19D5.11c	ctf8	Ctf18 RFC-like complex subunit Ctf8
65	SPAC19E9.02	fin1	serine/threonine protein kinase, NIMA related Fin1
66	SPAC19G12.13c	poz1	shelterin complex subunit Poz1
67	SPAC19G12.17	erh1	enhancer of rudimentary homolog Erh1
68	SPAC1B3.17	clr2	chromatin silencing protein Clr2
69	SPAC1D4.09c	rtf2	replication termination factor Rtf2
70	SPAC1D4.11c	lkh1	dual specificity protein kinase Lkh1
71	SPAC1F3.01	rrp6	exosome 3'-5' exoribonuclease subunit Rrp6
72	SPAC1F3.06c	spo15	mitotic and mieotic spindle pole body protein Spo15

73	SPAC1F7.01c	spt6	nucleosome remodeling protein Spt6
74	SPAC20G4.04c	hus1	checkpoint clamp complex protein Hus1
75	SPAC20G8.08c	fft1	SMARCAD1 family ATP-dependent DNA helicase Fft1 (predicted)
76	SPAC20H4.03c	tfs1	general transcription elongation factor TFIIS
77	SPAC20H4.10	ufd2	ubiquitin-protein ligase E4 Ufd2 (predicted)
78	SPAC21E11.03c	pcr1	transcription factor Pcr1
79	SPAC21E11.05c	cyp8	cyclophilin family peptidyl-prolyl cis-trans isomerase Cyp8
80	SPAC222.04c	ies6	Ino80 complex subunit Ies6
81	SPAC222.15	meu13	Tat binding protein 1(TBP-1)-interacting protein (TBPIP) homolog (predicted)
82	SPAC22A12.01c	pso2	DNA 5' exonuclease (predicted)
83	SPAC22E12.11c	set3	histone lysine methyltransferase Set3
84	SPAC22E12.19	snt1	Set3 complex subunit Snt1
85	SPAC22F3.02	atf31	transcription factor Atf31
86	SPAC22F3.09c	res2	MBF transcription factor complex subunit Res2
87	SPAC22F8.12c	shf1	small histone ubiquitination factor Shf1
88	SPAC22H12.02	tfg3	TFIID, TFIIF, Ino80, SWI/SNF, and NuA3 complex subunit Tfg3
89	SPAC23A1.07		ubiquitin-protein ligase E3 (predicted)
90	SPAC23C11.08	php3	CCAAT-binding factor complex subunit Php3
91	SPAC23C11.15	pst2	Clr6 histone deacetylase complex subunit Pst2
92	SPAC23C4.03	hrk1	haspin related kinase Hrk1
93	SPAC23D3.01	pdp3	PWWP domain protein, involved in chromatin remodeling (predicted)
94	SPAC23D3.09	arp42	SWI/SNF and RSC complex subunit Arp42
95	SPAC23E2.01	fep1	iron-sensing transcription factor Fep1
96	SPAC23E2.03c	ste7	arrestin family meiotic suppressor protein Ste7
97	SPAC23G3.04	ies4	Ino80 complex subunit Ies4
98	SPAC23G3.07c	snf30	SWI/SNF complex subunit Snf30
99	SPAC23G3.08c	ubp7	ubiquitin C-terminal hydrolase Ubp7
100	SPAC23G3.10c	ssr3	SWI/SNF and RSC complex subunit Ssr3
101	SPAC23H3.05c	swd1	Set1C complex subunit Swd1
102	SPAC23H4.12	alp13	MRG family Clr6 histone deacetylase complex subunit Alp13
103	SPAC24B11.10c	cfh1	SEL1/TPR repeat protein Cfh1 (predicted)
104	SPAC25A8.01c	fft3	SMARCAD1 family ATP-dependent DNA helicase Fft3
105	SPAC25H1.02	jmj1	histone demethylase Jmj1 (predicted)
106	SPAC26H5.03	pcf2	CAF assembly factor (CAF-1) complex subunit B, Pcf2
107	SPAC29A4.09		rRNA exonuclease Rrp17 (predicted)
108	SPAC29A4.18	prw1	Clr6 histone deacetylase complex subunit Prw1
109	SPAC29B12.02c	set2	histone lysine H3-K36 methyltransferase Set2
110	SPAC29B12.03	spd1	ribonucleotide reductase (RNR) inhibitor
111	SPAC29B12.06c	rcd1	CCR4-Not complex RNA-binding protein subunit Rcd1
112	SPAC29B12.08	clr5	Clr5 protein
113	SPAC2C4.07c	dis32	3'-5'-exoribonuclease activity Dis3L2
114	SPAC2F3.15	lsk1	P-TEFb-associated cyclin-dependent protein kinase Lsk1
115	SPAC2F3.16		ubiquitin-protein ligase E3, implicated in DNA repair (predicted)
116	SPAC2F7.07c	cph2	Clr6 histone deacetylase associated PHD protein Cph2
117	SPAC2F7.08c	snf5	SWI/SNF complex subunit Snf5
118	SPAC2G11.05c	rim20	BRO1 domain protein Rim20
119	SPAC2G11.10c	uba42	thiosulfate sulfurtransferase, URM1 activating enzyme E1-type Uba42 (predicted)
120	SPAC30D11.07	nth1	DNA endonuclease III
121	SPAC31A2.09c	apm4	AP-2 adaptor complex mu subunit Apm4 (predicted)
122	SPAC31A2.16	gef2	RhoGEF Gef2
123	SPAC31G5.09c	spk1	MAP kinase Spk1
124	SPAC31G5.19	abo1	ATPase with bromodomain protein
125	SPAC323.03c		Schizosaccharomyces specific protein
126	SPAC328.05	hrb1	RNA-binding protein involved in export of mRNAs Hrb1 (predicted)
127	SPAC32A11.03c	phx1	stationary phase-specific homeobox transcription factor Phx1
128	SPAC343.04c	gid7	GID complex subunit Gid7 (predicted)
129	SPAC343.11c	msc1	Swr1 complex subunit Msc1
130	SPAC343.18	rfp2	SUMO-targeted ubiquitin-protein ligase subunit Rfp2
131	SPAC3A11.05c	kms1	meiotic spindle pole body KASH domain protein Kms1
132	SPAC3C7.08c	elf1	AAA family ATPase Elf1
133	SPAC3F10.10c	map3	pheromone M-factor receptor Map3
134	SPAC3F10.12c		transcription factor (predicted)
135	SPAC3G6.01	hrp3	ATP-dependent DNA helicase Hrp3
136	SPAC3G6.06c	rad2	FEN-1 endonuclease Rad2
137	SPAC3G6.11	chl1	ATP-dependent DNA helicase Chl1 (predicted)
138	SPAC3G9.07c	hos2	histone deacetylase (class I) Hos2
139	SPAC3H1.11	hsr1	transcription factor Hsr1
140	SPAC3H1.12c	snt2	Lid2 complex PHD finger subunit Snt2
141	SPAC3H8.08c		transcription factor (predicted)
142	SPAC4A8.09c	cwf21	complexed with Cdc5 protein Cwf21
143	SPAC4F8.11	sea2	SEA complex WD repeat subunit Sea2 (predicted)
144	SPAC4G9.06c	chz1	histone H2A-H2B dimer chaperone Chz1 (predicted)
145	SPAC4H3.02c	swc3	Swr1 complex subunit Swc3
146	SPAC4H3.05	srs2	ATP-dependent DNA helicase, UvrD subfamily

147	SPAC56F8.16	esc1	transcription factor Esc1 (predicted)
148	SPAC57A10.09c	nhp6	High-mobility group non-histone chromatin protein (predicted)
149	SPAC5D6.02c	mug165	Clr6 histone deacetylase complex subunit Mug165
150	SPAC5D6.08c	mes1	meiotic APC inhibitor Mes1
151	SPAC630.14c	tup12	transcriptional corepressor Tup12
152	SPAC631.02	bdf2	BET family double bromodomain protein Bdf2
153	SPAC637.09	rex1	3'-5'- exoribonuclease Rex1 (predicted)
154	SPAC644.14c	rad51	RecA family recombinase Rad51/Rhp51
155	SPAC664.01c	swi6	heterochromatin (HP1) family chromodomain protein Swi6
156	SPAC664.02c	arp8	Ino80 complex actin-like protein Arp8
157	SPAC664.07c	rad9	checkpoint clamp complex protein Rad9
158	SPAC664.15	caf4	CCR4-Not complex subunit Caf4/Mdv1 (predicted)
159	SPAC688.06c	slx4	structure-specific endonuclease subunit Slx4
160	SPAC694.06c	mrc1	claspin, Mrc1
161	SPAC6B12.05c	ies2	Ino80 complex subunit Ies2
162	SPAC6B12.07c		ubiquitin-protein ligase E3 with SPX domain, human LORNRF1 ortholog (predicted)
163	SPAC6B12.14c		conserved fungal protein
164	SPAC6B12.16	meu26	DUF4451 family conserved fungal protein
165	SPAC6F12.09	rdp1	RNA-directed RNA polymerase Rdp1
166	SPAC6F6.09	eaf6	Mst2/NuA4 histone acetyltransferase complex subunit Eaf6
167	SPAC6G9.03c	mug183	histone H3.3 H4 heterotetramer chaperone Rtt106-like (predicted)
168	SPAC6G9.10c	sen1	ATP-dependent 5' to 3' DNA/RNA helicase Sen1
169	SPAC6G9.16c	xrc4	XRCC4 nonhomologous end joining factor Xrc4
170	SPAC7D4.04	atg11	autophagy associated protein Atg11
171	SPAC7D4.14c	iss10	NURS complex subunit Iss10
172	SPAC821.07c	moc3	transcription factor Moc3
173	SPAC823.03	ppk15	serine/threonine protein kinase Ppk15 (predicted)
174	SPAC824.04	swd22	mRNA cleavage and polyadenylation specificity factor complex, WD repeat protein Swd22
175	SPAC890.07c	rmt1	type I protein arginine N-methyltransferase Rmt1
176	SPAC8C9.14	prr1	transcription factor Prr1
177	SPAC8C9.17c	spc34	DASH complex subunit Spc34
178	SPAC8F11.03	msh3	MutS protein homolog 3
179	SPAC9E9.08	rad26	ATRIP, ATR checkpoint kinase regulatory subunit Rad26
180	SPAC9E9.10c	cbh1	CENP-B homolog Cbh1
181	SPAP14E8.02	tos4	chromatin binding FHA domain protein Tos4 (predicted)
182	SPAP27G11.15	slx1	structure-specific endonuclease catalytic subunit Slx1
183	SPAP32A8.03c	bop1	ubiquitin-protein ligase E3, human RNF126 ortholog (predicted)
184	SPAP8A3.02c	ofd2	histone H2A dioxygenase Ofd2
185	SPAPB1E7.02c	mcl1	DNA polymerase alpha accessory factor Mcl1
186	SPAPB24D3.01	toe3	transcription factor (predicted)
187	SPAPB2B4.03	cig2	G1/S-specific B-type cyclin Cig2
188	SPBC1105.04c	cbp1	CENP-B homolog
189	SPBC119.08	pmk1	MAP kinase Pmk1
190	SPBC119.14	rti1	Rad22 homolog Rti1
191	SPBC1198.11c	reb1	RNA polymerase I transcription termination factor/ RNA polymerase II transcription factor Reb1
192	SPBC11B10.05c	rsp1	random septum position protein, DNAJ domain protein Rsp1
193	SPBC11B10.08		WW domain containing conserved fungal protein
194	SPBC11B10.10c	pht1	histone H2A variant H2A.Z, Pht1
195	SPBC1347.07	rex2	RNA exonuclease (predicted)
196	SPBC13E7.08c	leo1	RNA polymerase II associated Paf1 complex subunit Leo1
197	SPBC13G1.08c	ash2	Ash2-trithorax family protein
198	SPBC146.06c	fan1	DNA repair protein Fan1
199	SPBC14C8.17c	spt8	SAGA complex subunit Spt8
200	SPBC14F5.07	doa10	ER ubiquitin-protein ligase E3 Doa10 (predicted)
201	SPBC15C4.01c	oca3	TPR repeat protein Oca3/ ER membrane protein complex Ecm2 (predicted)
202	SPBC15C4.06c		ubiquitin-protein ligase E3 Meu34, human RNF13 family homolog, unknown biological role (predicted)
203	SPBC15D4.03	slm9	histone H3.3 H4 chaperone, hira family Slm9
204	SPBC1604.09c	rex4	exoribonuclease Rex4 (predicted)
205	SPBC1604.16c		RNA-binding protein, G-patch type, human GPANK1 ortholog
206	SPBC1685.08	cti6	histone deacetylase complex ubiquitin-like protein ligase subunit Cti6
207	SPBC16A3.07c	nrm1	MBF complex corepressor Nrm1
208	SPBC16A3.19	eaf7	histone acetyltransferase complex subunit Eaf7
209	SPBC16D10.07c	sir2	Sirtuin family histone deacetylase Sir2
210	SPBC16E9.11c	pub3	HECT-type ubiquitin-protein ligase E3 Pub3 (predicted)
211	SPBC16E9.12c	pab2	poly(A) binding protein Pab2
212	SPBC16G5.03	mrz1	ubiquitin-protein ligase E3/SUMO transferase, Topors, possibly associated with DNA damage (predicted)
213	SPBC16G5.15c	fkh2	forkhead transcription factor Fkh2
214	SPBC16G5.17		transcription factor, zf-fungal binuclear cluster type (predicted)
215	SPBC1703.04	mlh1	MutL family protein Mlh1 (predicted)
216	SPBC1703.14c	top1	DNA topoisomerase I
217	SPBC1709.11c	png2	ING family histone acetyltransferase complex PHD-type zinc finger subunit Png2
218	SPBC1711.14	rec15	meiotic recombination protein Rec15
219	SPBC1718.02	hop1	linear element associated protein Hop1
220	SPBC1734.06	rhp18	Rad18 homolog ubiquitin protein ligase E3, Rhp18

221	SPBC1734.15	rsc4	RSC complex subunit Rsc4
222	SPBC1773.16c		transcription factor, zf-fungal binuclear cluster type(predicted)
223	SPBC1778.10c	ppk21	serine/threonine protein kinase Ppk21 (predicted)
224	SPBC17D11.04c	nto1	histone acetyltransferase complex PHD finger subunit Nto1 (predicted)
225	SPBC17G9.05	rct1	cyclophilin family peptidyl-prolyl cis-trans isomerase, RRM-containing Rct1
226	SPBC18H10.06c	swd2	Set1C complex subunit Swd2.1
227	SPBC18H10.15	cdk11	serine/threonine protein kinase Cdk11
228	SPBC19C7.02	ubr1	N-end-recognizing protein, UBR ubiquitin-protein ligase E3 Ubr1
229	SPBC1A4.03c	top2	DNA topoisomerase II
230	SPBC1D7.03	clg1	cyclin-like protein involved in autophagy Clg1 (predicted)
231	SPBC1D7.04	mlo3	RNA binding protein Mlo3
232	SPBC20F10.05	nrl1	RNAi-mediated silencing protein, human NRDE2 ortholog Nrl1
233	SPBC20F10.10	psl1	cyclin pho85 family Psl1 (predicted)
234	SPBC215.03c	csn1	COP9/signalosome complex subunit Csn1
235	SPBC215.06c		nucleolar RNA-binding protein, human LYAR homolog, implicated in transcriptional regulation
236	SPBC215.07c	pdp2	PWWP domain protein Pdp2 (predicted)
237	SPBC216.05	rad3	ATR checkpoint kinase Rad3
238	SPBC216.06c	swi1	replication fork protection complex subunit Swi1
239	SPBC21B10.13c	yox1	MBF complex corepressor Yox1
240	SPBC21C3.02c	dep1	Sds3-like family protein Dep1
241	SPBC21C3.20c	git1	C2 domain protein Git1
242	SPBC21D10.09c	rkr1	RQC complex ubiquitin-protein ligase E3 Rkr1 (predicted)
243	SPBC21D10.10	bdc1	bromodomain protein Bdc1
244	SPBC23E6.02	rrp2	ATP-dependent DNA helicase/ ubiquitin-protein ligase E3 (predicted)
245	SPBC23E6.09	ssn6	transcriptional corepressor Ssn6
246	SPBC23G7.13c		plasma membrane urea transmembrane transporter (predicted)
247	SPBC24C6.05	sec28	coatomer epsilon subunit (predicted)
248	SPBC25B2.08		Schizosaccharomyces pombe specific protein
249	SPBC26H8.09c	snf59	SWI/SNF complex subunit Snf59
250	SPBC28E12.02		RNA-binding protein
251	SPBC28F2.07	sfr1	Swi five-dependent recombination mediator Sfr1
252	SPBC28F2.10c	ngg1	SAGA complex subunit Ngg1/Ada3
253	SPBC29A10.03c	pcf1	CAF assembly factor (CAF-1) complex large subunit Pcf1
254	SPBC29A10.05	exo1	exonuclease I Exo1
255	SPBC29A10.14	rec8	meiotic cohesin complex subunit Rec8
256	SPBC29A3.03c	gid2	GID complex ubiquitin-protein ligase E3 subunit Gid2/Rmd5 (predicted)
257	SPBC29A3.05	vps71	Swr1 complex subunit Vps71
258	SPBC29A3.13	pdp1	PWWP domain protein Pdp1
259	SPBC29B5.01	atf1	transcription factor, Atf-CREB family Atf1
260	SPBC2A9.04c	san1	sir antagonist, ubiquitin-protein ligase E3
261	SPBC2D10.11c	nap2	histone H2A-H2B chaperone Nap2
262	SPBC2D10.17	clr1	SHREC complex intermodule linker subunit Clr1
263	SPBC2F12.09c	atf21	transcription factor, Atf-CREB family Atf21
264	SPBC2F12.12c	cay1	cactin, spliceosome complex subunit
265	SPBC2G2.06c	apl1	AP-2 adaptor complex beta subunit Apl1 (predicted)
266	SPBC2G2.14	csi1	mitotic centromere-SPB clustering protein Csi1
267	SPBC2G5.02c	ckb2	CK2 family regulatory subunit Ckb2 (predicted)
268	SPBC30B4.04c	sol1	SWI/SNF complex subunit Sol1
269	SPBC30D10.10c	tor1	serine/threonine protein kinase Tor1
270	SPBC31F10.07	lsb5	actin cortical patch component Lsb5 (predicted)
271	SPBC31F10.10c	mub1	Armadillo-type fold protein, zf-MYND type zinc finger protein, Mub1-Rad6-Ubr2 ubiquitin ligase complex Mub1 (predicted)
272	SPBC31F10.13c	hip1	histone H3.3 H4 chaperone, hira family Hip1
273	SPBC31F10.14c	hip3	HIRA interacting protein Hip3
274	SPBC32F12.07c		membrane associated ubiquitin-protein ligase E3, MARCH family (predicted)
275	SPBC32H8.06	mug93	TPR repeat protein, meiotically spliced
276	SPBC337.03	rhn1	RNA polymerase II transcription termination factor homolog
277	SPBC342.05	crb2	DNA repair protein Rad9 homolog Crb2
278	SPBC342.06c	rtt109	RTT109 family histone lysine acetyltransferase
279	SPBC354.03	swd3	WD repeat protein Swd3
280	SPBC354.05c	sre2	membrane-tethered transcription factor Sre2
281	SPBC365.10	arp5	Ino80 complex actin-like protein Arp5
282	SPBC36B7.05c	pib1	endosomal and vacuolar ubiquitin-protein ligase E3/phosphatidylinositol(3)-phosphate binding protein Pib1
283	SPBC36B7.08c	ccp1	histone chaperone, CENP-A nucleosome disassembly Ccp1
284	SPBC3B8.02	php5	CCAAT-binding factor complex subunit Php5
285	SPBC3D6.04c	mad1	mitotic spindle checkpoint protein Mad1
286	SPBC3D6.09	dpb4	DNA polymerase epsilon subunit Dpb4
287	SPBC3H7.13	far10	SIP/FAR complex FHA domain subunit Far10/Csc1
288	SPBC4.05	mlo2	ubiquitin protein ligase E3 component human N-recognin 7 homolog Mlo2
289	SPBC428.06c	rxt2	histone deacetylase complex subunit Rxt2
290	SPBC428.08c	clr4	histone lysine H3 methyltransferase Clr4
291	SPBC4B4.03	rsc1	RSC complex subunit Rsc1
292	SPBC4C3.12	sep1	forkhead transcription factor Sep1
293	SPBC530.08		membrane-tethered transcription factor (predicted)
294	SPBC530.14c	dsk1	SR protein-specific kinase Dsk1

295	SPBC543.07	pek1	MAP kinase kinase Pek1
296	SPBC56F2.03	arp10	dynactin complex actin-like protein Arp10 (predicted)
297	SPBC56F2.05c		transcription factor (predicted)
298	SPBC582.04c	dsh1	RNAi protein, Dsh1
299	SPBC582.06c	mcp6	horsetail movement protein Hrs1/Mcp6
300	SPBC609.05	pob3	histone H2A-H2B chaperone, FACT complex subunit Pob3
301	SPBC651.11c	apm3	AP-3 adaptor complex subunit Apm3 (predicted)
302	SPBC660.06		WW domain containing conserved fungal protein
303	SPBC660.14	mik1	mitotic inhibitor kinase Mik1
304	SPBC6B1.04	mde4	microtubule-site clamp monopolin complex subunit Mde4
305	SPBC6B1.06c	ubp14	Lys48-specific deubiquitinase Ubp14
306	SPBC725.02	mpr1	histidine-containing response regulator phosphotransferase Mpr1
307	SPBC725.11c	php2	CCAAT-binding factor complex subunit Php2
308	SPBC776.02c	dis2	serine/threonine protein phosphatase PP1, Dis2
309	SPBC776.16	mis20	centromere protein Mis20/Eic2
310	SPBC800.03	clr3	histone deacetylase (class II) Clr3
311	SPBC83.03c	tas3	RITS complex subunit 3
312	SPBC902.02c	ctf18	Ctf18 RFC-like complex subunit Ctf18
313	SPBC902.04	rmn1	RNA-binding protein
314	SPBC902.06	mto2	gamma tubulin complex linker Mto2
315	SPBP16F5.03c	tra1	SAGA complex phosphatidylinositol pseudokinase Tra1
316	SPBP22H7.05c	abo2	ATPase with bromodomain protein (predicted)
317	SPBP23A10.05	ssr4	SWI/SNF and RSC complex subunit Ssr4
318	SPBP35G2.08c	air1	TRAMP complex zinc knuckle subunit Air1
319	SPBP35G2.10	mit1	SHREC complex ATP-dependent DNA helicase subunit Mit1
320	SPBP35G2.13c	swc2	Swr1 complex subunit Swc2
321	SPBP8B7.07c	set6	histone lysine methyltransferase Set6 (predicted)
322	SPBP8B7.23	rnf10	ubiquitin-protein ligase E3 (predicted)
323	SPBP8B7.28c	stc1	CLRC ubiquitin ligase complex linker protein, LIM-like Stc1
324	SPCC1020.12c	xap5	xap-5-like protein
325	SPCC11E10.08	rik1	CLRC ubiquitin ligase complex WD repeat protein Rik1
326	SPCC1223.13	cbf12	CBF1/Su(H)/LAG-1 family transcription factor Cbf12
327	SPCC1235.05c	fft2	SMARCAD1 family ATP-dependent DNA helicase Fft2 (predicted)
328	SPCC1235.09	hif2	Set3 complex subunit Hif2
329	SPCC1235.12c	mug146	Schizosaccharomyces specific protein Mug46
330	SPCC1259.04	iec3	Ino80 complex subunit Iec3
331	SPCC1259.07	rxt3	transcriptional regulatory protein Rxt3
332	SPCC126.02c	pku70	Ku domain protein Pku70
333	SPCC126.04c	sgf73	SAGA complex deubiquitinating submodule subunit Sgf73
334	SPCC126.07c	asr1	ubiquitin-protein ligase E3 Asr1 (predicted)
335	SPCC126.11c		RNA-binding protein, rrm type
336	SPCC126.13c	sap18	splicing factor Sap18 (predicted)
337	SPCC132.02	hst2	Sirtuin family histone deacetylase Hst2
338	SPCC1393.02c	spt2	non-specific DNA binding protein Spt2 (predicted)
339	SPCC1393.05	ers1	RNA-silencing factor Ers1
340	SPCC1442.13c	sqs2	R3H and G-patch domain protein Sqs2
341	SPCC1450.02	bdf1	Swr1 complex bromodomain subunit Bdf1
342	SPCC1450.03	utp502	ribonucleoprotein (RNP) complex Utp502 (predicted)
343	SPCC1494.03	arz1	human RAP1 GTPase-GDP dissociation stimulator ortholog, Zfs1 target number 1
344	SPCC162.11c	urk1	uridine kinase/uracil phosphoribosyltransferase (predicted)
345	SPCC1620.14c	snf22	ATP-dependent DNA helicase Snf22
346	SPCC1682.13	laf2	Clr6 associated factor 2, Laf2
347	SPCC16C4.11	pef1	Pho85/PhoA-like cyclin-dependent kinase Pef1
348	SPCC1739.03	hrr1	Helicase Required for RNAi-mediated heterochromatin assembly Hrr1
349	SPCC1739.05	set5	histone lysine methyltransferase Set5 (predicted)
350	SPCC1739.07	cti1	exosome C1D family subunit Cti1
351	SPCC1739.12	ppe1	serine/threonine protein phosphatase Ppe1
352	SPCC1753.03c	rec7	meiotic recombination protein Rec7
353	SPCC1840.04	pca1	metacaspase Pca1
354	SPCC188.07	ccq1	shelterin complex HEAT repeat subunit Ccq1
355	SPCC188.13c	dcr1	dicer
356	SPCC18B5.03	wee1	M phase inhibitor protein kinase Wee1
357	SPCC18B5.07c	nup61	nucleoporin Nup61
358	SPCC24B10.07	gad8	AGC family protein kinase Gad8
359	SPCC24B10.08c	ada2	SAGA complex subunit Ada2
360	SPCC24B10.14c	xlf1	XRCC4-like nonhomologous end joining factor, Cernunnon Xlf1/Nej1
361	SPCC24B10.19c	nts1	Clr6 histone deacetylase complex subunit Nts1
362	SPCC297.03	ssp1	Ca2+/calmodulin-dependent (CaMMK)-like protein kinase Ssp1
363	SPCC297.04c	set7	histone lysine H3-K37 methyltransferase Set7
364	SPCC2H8.05c	dbl1	double strand break localizing Dbl1
365	SPCC306.04c	set1	histone lysine H3-K4 methyltransferase Set1
366	SPCC31H12.08c	ccr4	CCR4-Not complex 3'-5'-exoribonuclease subunit Ccr4
367	SPCC330.01c	rhp16	Rad16 homolog ATP-dependent DNA helicase/ ubiquitin protein ligase E3 Rhp16
368	SPCC330.02	rhp7	Rad7 homolog Rhp7

369	SPCC338.16	pof3	F-box protein Pof3
370	SPCC364.02c	bis1	splicing factor Bis1
371	SPCC364.06	nap1	histone H2A-H2B chaperone Nap1
372	SPCC417.07c	mto1	gamma tubulin complex linker Mto1
373	SPCC417.09c		transcription factor (predicted)
374	SPCC4B3.12	set9	histone lysine H4-K20 methyltransferase Set9
375	SPCC4G3.15c	not2	CCR4-Not complex NOT box subunit Not2
376	SPCC4G3.19	alp16	gamma tubulin complex subunit Alp16
377	SPCC548.05c	dbl5	ubiquitin-protein ligase E3 Dbl5
378	SPCC550.12	arp6	actin-like protein Arp6
379	SPCC550.15c	rei1	ribosome biogenesis protein Rei1 (predicted)
380	SPCC553.04	cyp9	WD repeat containing cyclophilin family peptidyl-prolyl cis-trans isomerase Cyp9 (predicted)
381	SPCC576.13	swc5	Swr1 complex subunit Swc5
382	SPCC594.05c	spf1	Set1C ubiquitin-protein ligase E3 subunit Spf1
383	SPCC61.02	spt3	SAGA complex subunit Spt3
384	SPCC613.12c	raf1	CLRC ubiquitin ligase complex WD repeat subunit Raf1/Dos1
385	SPCC622.15c		Schizosaccharomyces specific protein
386	SPCC622.16c	epe1	Jmjc domain chromatin associated protein Epe1
387	SPCC622.19	jmj4	peptidyl-lysine 3-dioxygenase activity jmj4 (predicted)
388	SPCC645.13	bye1	transcription elongation regulator Bye1 (predicted)
389	SPCC663.11	saf1	splicing associated factor Saf1
390	SPCC663.12	cid12	poly(A) polymerase Cid12
391	SPCC736.08	cbf11	CBF1/Su(H)/LAG-1 family transcription factor Cbf11
392	SPCC736.11	ago1	argonaute
393	SPCC757.09c	rnc1	KH domain RNA-binding protein Rnc1
394	SPCC895.06	elp2	elongator complex WD repeat protein Elp2 (predicted)
395	SPCC895.07	alp14	TOG/XMAP215 microtubule plus end tracking polymerase Alp14
396	SPCC970.07c	raf2	CLRC ubiquitin ligase complex subunit Raf2
