# Supplementary material for: Local chromatin context regulates the genetic requirements of the heterochromatin spreading reaction
Source: PLoS Genet. 2022 May 18;18(5):e1010201. doi: 10.1371/journal.pgen.1010201 (PMC9154106; doi:10.1371/journal.pgen.1010201)
Supplement: S2 Table — List of S. pombe strains used in this study. (PDF) [file pgen.1010201.s017.pdf]

S2 Table: Strain table

Strain	Genotype
PAS075	Locus2*::ade6p::3xE2C:hygMX* at Locus2 (between SPBC1711.11 and SPBC1711.12)
PM003	Wild-type strain: h(+); ura4-D18; leu1-32; ade6-M216; his7-366
PM006	972 h- wild-type
PAS193	Δ*K*::*ade6p:*mKO2; *ade6p:*SF-GFP between *REIII* and mat3M; *ade6p*:3xE2C*: hygMX* at Locus2; *clr4::kanMX,* h(-)
PAS216	*cenH*::*ade6p:*SF-GFP(Kint2); *mat3m(Eco*RV):: *ade6p:*mKO2; *ade6p:*3xE2C: *hygMX* at Locus2; *clr4::kanMX,* h90
PAS217	*cenH*: *ade6p:*SF-GFP (Kint2); *mat3m(Eco*RV):: *ade6p:*mKO2; *ade6p:*3xE2C: *hygMX* at Locus2, h90
PAS231	*ura4::natMX:dh:ade6p:*SF-GFP, *ade6p:*mKO2 3 kb, *leu1*::*ade6p:*3xE2C: *hygMX*
PAS331	*cenH*:: *ade6p:*SF-GFP (Kint2); *mat3m(Eco*RV):: *ade6p:*mKO2; *ade6p:*3xE2C:*hygMX* at Locus2; Δ*REIII*::REIII(Δs1, Δs2) in *clr4::kanMX,* h90
PAS332	*cenH*:: *ade6p:*SF-GFP (Kint2); *mat3m(Eco*RV):: *ade6p:*mKO2; *ade6p:*3xE2C:*hygMX* at Locus2; Δ*REIII*::REIII(Δs1, Δs2), h90
PAS353	*ura4::natMX:dh:ade6p:*SF-GFP, *ade6p:*mKO2 3 kb, *leu1*::*ade6p:*3xE2C: *hygMX; gcn5::kanMX*
PAS482	Δ*K*::*ade6p:*mKO2; *ade6p:* SF-GFP between *REIII* and mat3M; *ade6p:*3xE2C*: hygMX* at Locus2, h(-); 'OFF' allele
PAS795	*cenH*: *ade6p:*SF-GFP (Kint2); *mat3m(Eco*RV):: *ade6p:*mKO2; *ade6p:*3xE2C: *hygMX* at Locus2; *fkh2::natMX*
PAS796	*cenH*: *ade6p:*SF-GFP (Kint2); *mat3m(Eco*RV):: *ade6p:*mKO2; *ade6p:*3xE2C: *hygMX* at Locus2; *prw1::kanMX*
PAS797	*cenH*: *ade6p:*SF-GFP (Kint2); *mat3m(Eco*RV):: *ade6p:*mKO2; *ade6p:*3xE2C: *hygMX* at Locus2; *png3::kanMX*
PAS798	*cenH*:: *ade6p:*SF-GFP (Kint2); *mat3m(Eco*RV):: *ade6p:*mKO2; *ade6p:*3xE2C:*hygMX* at Locus2; Δ*REIII*::REIII(Δs1, Δs2);*fkh2::natMX*
PAS799	*cenH*:: *ade6p:*SF-GFP (Kint2); *mat3m(Eco*RV):: *ade6p:*mKO2; *ade6p:*3xE2C:*hygMX* at Locus2; Δ*REIII*::REIII(Δs1, Δs2); *prw1::kanMX*
PAS800	*cenH*:: *ade6p:*SF-GFP (Kint2); *mat3m(Eco*RV):: *ade6p:*mKO2; *ade6p:*3xE2C:*hygMX* at Locus2; Δ*REIII*::REIII(Δs1, Δs2); *png3::kanMX*
PAS803	Δ*K*::*ade6p:*mKO2; *ade6p:* SF-GFP between *REIII* and mat3M; *ade6p:*3xE2C*: hygMX* at Locus2; 'OFF' allele; *fkh2::natMX*
PAS808	*cenH*: *ade6p:*SF-GFP (Kint2); *mat3m(Eco*RV):: *ade6p:*mKO2; *ade6p:*3xE2C: *hygMX* at Locus2; *fkh2::natMX; prw1::kanMX* by cross
PAS809	*cenH*: *ade6p:*SF-GFP (Kint2); *mat3m(Eco*RV):: *ade6p:*mKO2; *ade6p:*3xE2C: *hygMX* at Locus2; *fkh2::natMX; prw1::kanMX* by sequential knockout
PAS810	*cenH*:: *ade6p:*SF-GFP (Kint2); *mat3m(Eco*RV):: *ade6p:*mKO2; *ade6p:*3xE2C:*hygMX* at Locus2; Δ*REIII*::REIII(Δs1, Δs2); *fkh2::natMX; prw1::kanMX* by cross
PAS811	*cenH*:: *ade6p:*SF-GFP (Kint2); *mat3m(Eco*RV):: *ade6p:*mKO2; *ade6p:*3xE2C:*hygMX* at Locus2; Δ*REIII*::REIII(Δs1, Δs2); *fkh2::natMX; prw1::kanMX* by sequential knockout
PAS813	*cenH*:: *ade6p:*SF-GFP (Kint2); *mat3m(Eco*RV):: *ade6p:*mKO2; *ade6p:*3xE2C:*hygMX* at Locus2; Δ*REIII*::REIII(Δs1, Δs2); *apm3::natMX*
PAS816	apl5:SF-GFP:*hygMX*; Swi6:E2C:*kanMX*
PAS817	apm3:SF-GFP:*hygMX;* Swi6:E2C:*kanMX*
PAS833	fkh2::Fkh2:13XMYC Zilio et al.
PAS836	clr6::Clr6:13XMYC:*hygMX;* fkh2::Fkh2:TAP:*kanMX* Zilio et al.

PAS838	fkh2::Fkh2:13XMYC:*hygMX;* sds3::Sds3:TAP:*kanMX* Zilio et al.
PAS839	*cenH*:: *ade6p:*SF-GFP (Kint2); *mat3m(Eco*RV):: *ade6p:*mKO2; *ade6p:*3xE2C:*hygMX* at Locus2; Δ*REIII*::REIII(Δs1, Δs2); *clr6-1,* h90
PAS841	*cenH*:: *ade6p:*SF-GFP (Kint2); *mat3m(Eco*RV):: *ade6p:*mKO2; *ade6p:*3xE2C:*hygMX* at Locus2; Δ*REIII*::REIII(Δs1, Δs2), fkh2::Fkh2:13XMYC:*hygMX*, h90
PAS842	*cenH*:: *ade6p:*SF-GFP (Kint2); *mat3m(Eco*RV):: *ade6p:*mKO2; *ade6p:*3xE2C:*hygMX* at Locus2; Δ*REIII*::REIII(Δs1, Δs2); *clr3-D232N:natMX,* h90
PAS850	*cenH*:: *ade6p:*SF-GFP (Kint2); *mat3m(Eco*RV):: *ade6p:*mKO2; *ade6p:*3xE2C:*hygMX* at Locus2; Δ*REIII*::REIII(Δs1, Δs2), fkh2::Fkh2:13XMYC:*hygMX*; *prw1::kanMX,* h90
PAS867	*cenH*:: *ade6p:*SF-GFP (Kint2); *mat3m(Eco*RV):: *ade6p:*mKO2; *ade6p:*3xE2C:*kanMX* at Locus2; Δ*REIII*::REIII(Δs1, Δs2), clr6::Clr6:13XMYC:*hygMX,* h90
PAS868	*cenH*:: *ade6p:*SF-GFP (Kint2); *mat3m(Eco*RV):: *ade6p:*mKO2; *ade6p:*3xE2C:*kanMX* at Locus2; Δ*REIII*::REIII(Δs1, Δs2), clr6::Clr6:13XMYC:*hygMX; fkh2::natMX,* h90
PAS869	*cenH*:: *ade6p:*SF-GFP (Kint2); *mat3m(Eco*RV):: *ade6p:*mKO2; *ade6p:*3xE2C:*kanMX* at Locus2; Δ*REIII*::REIII(Δs1, Δs2), *apl5::kanMX; apm3::natMX,* h90
PAS870	*cenH*:: *ade6p:*SF-GFP (Kint2); *mat3m(Eco*RV):: *ade6p:*mKO2; *ade6p:*3xE2C:*kanMX* at Locus2; Δ*REIII*::REIII(Δs1, Δs2), *apl5::kanMX,* h90
PAS889	*cenH*:: *ade6p:*SF-GFP (Kint2); *mat3m(Eco*RV):: *ade6p:*mKO2; *ade6p:*3xE2C:*kanMX* at Locus2; *saf5::kanMX,* h90
PAS890	*cenH*:: *ade6p:*SF-GFP (Kint2); *mat3m(Eco*RV):: *ade6p:*mKO2; *ade6p:*3xE2C:*kanMX* at Locus2; *eaf6::kanMX,* h90
PAS891	*cenH*:: *ade6p:*SF-GFP (Kint2); *mat3m(Eco*RV):: *ade6p:*mKO2; *ade6p:*3xE2C:*kanMX* at Locus2; *pht1::kanMX,* h90
PAS892	*cenH*:: *ade6p:*SF-GFP (Kint2); *mat3m(Eco*RV):: *ade6p:*mKO2; *ade6p:*3xE2C:*kanMX* at Locus2; *hip1::kanMX,* h90
PAS893	*cenH*:: *ade6p:*SF-GFP (Kint2); *mat3m(Eco*RV):: *ade6p:*mKO2; *ade6p:*3xE2C:*kanMX* at Locus2; *gad8::kanMX,* h90
PAS894	Δ*K*::*ade6p:*mKO2; *ade6p:* SF-GFP between *REIII* and mat3M; *ade6p:*3xE2C*: hygMX* at Locus2, h(-); *pht1::kanMX,* h90
PAS895	Δ*K*::*ade6p:*mKO2; *ade6p:* SF-GFP between *REIII* and mat3M; *ade6p:*3xE2C*: hygMX* at Locus2, h(-); *hip1::kanMX,* h90
PAS896	Δ*K*::*ade6p:*mKO2; *ade6p:* SF-GFP between *REIII* and mat3M; *ade6p:*3xE2C*: hygMX* at Locus2, h(-); *gad8::kanMX,* h90
PAS897	Δ*K*::*ade6p:*mKO2; *ade6p:* SF-GFP between *REIII* and mat3M; *ade6p:*3xE2C*: hygMX* at Locus2, h(-); *hip1::kanMX,* h90
PAS898	Δ*K*::*ade6p:*mKO2; *ade6p:* SF-GFP between *REIII* and mat3M; *ade6p:*3xE2C*: hygMX* at Locus2, h(-); *gad8::kanMX,* h90
PAS900	*ura4::natMX:dh:ade6p:*SF-GFP, *ade6p:*mKO2 3 kb, *leu1*::*ade6p:*3xE2C: *hygMX; eaf6::kanMX*
PAS901	*ura4::natMX:dh:ade6p:*SF-GFP, *ade6p:*mKO2 3 kb, *leu1*::*ade6p:*3xE2C: *hygMX; pht1::kanMX*
PAS902	*ura4::natMX:dh:ade6p:*SF-GFP, *ade6p:*mKO2 3 kb, *leu1*::*ade6p:*3xE2C: *hygMX; hip1::kanMX*
PAS903	*ura4::natMX:dh:ade6p:*SF-GFP, *ade6p:*mKO2 3 kb, *leu1*::*ade6p:*3xE2C: *hygMX; gad8::kanMX*
PAS904	*cenH*:: *ade6p:*SF-GFP (Kint2); *mat3m(Eco*RV):: *ade6p:*mKO2; *ade6p:*3xE2C:*kanMX* at Locus2; Δ*REIII*::REIII(Δs1, Δs2); *saf5::kanMX,* h90

PAS905	*cenH*:: *ade6p:*SF-GFP (Kint2); *mat3m(Eco*RV):: *ade6p:*mKO2; *ade6p:*3xE2C:*kanMX* at Locus2; Δ*REIII*::REIII(Δs1, Δs2); *eaf6::kanMX,* h90
PAS906	*cenH*:: *ade6p:*SF-GFP (Kint2); *mat3m(Eco*RV):: *ade6p:*mKO2; *ade6p:*3xE2C:*kanMX* at Locus2; Δ*REIII*::REIII(Δs1, Δs2); *pht1::kanMX,* h90
PAS907	*cenH*:: *ade6p:*SF-GFP (Kint2); *mat3m(Eco*RV):: *ade6p:*mKO2; *ade6p:*3xE2C:*kanMX* at Locus2; Δ*REIII*::REIII(Δs1, Δs2); *hip1::kanMX,* h90
PAS908	*cenH*:: *ade6p:*SF-GFP (Kint2); *mat3m(Eco*RV):: *ade6p:*mKO2; *ade6p:*3xE2C:*kanMX* at Locus2; Δ*REIII*::REIII(Δs1, Δs2); *gad8::kanMX,* h90
PAS932	smt-0 h(-); ura4-D18; leu1-32; ade6-M216; his7-366; *clr6-1*
PAS933	smt-0 h(-); ura4-D18; leu1-32; ade6-M216; his7-366; *prw1::kanMX*
