# Supplementary material for: Local chromatin context regulates the genetic requirements of the heterochromatin spreading reaction
Source: PLoS Genet. 2022 May 18;18(5):e1010201. doi: 10.1371/journal.pgen.1010201 (PMC9154106; doi:10.1371/journal.pgen.1010201)
Supplement: S3 Table — Primers for amplicons used in qPCR in this study. (PDF) [file pgen.1010201.s018.pdf]

S3 Table: Primers used for ChIP qPCR and RT qPCR

oligo	target	sequence
OAS 0367	orange F	AATTTTCCTGCTGATGGTCCT
OAS 0368	orange R	TGATCACCAGGCATTTCAAG
OAS 0846	green F	CTGCTGATAAGCAAAAGAACG
OAS 0847	green R	CCATATGATCACGCTTTTCG
OAS 1044	dg F	CCATCACCACTTTCATCTCC
OAS 1045	dg R	CAGGATACCTAGACGCACAA
OAS 1362	dh F	TTTAAAGCTTCACTACCATCGAAA
OAS 1363	dh R	TGCCAACAGTTTTTCCAACTT
OAS 1416	cenH right jxn F	ACCGGATGAATCTTCTCTTGAATA
OAS 1417	cenH right jxn R	TCCGTTTGTGTAATACTGAATGCT
OAS 1460	mat3-Mc DW F	ccggatagacattttgatgga
OAS 1461	mat3-Mc DW R	atgaaagccacagtgcacaa
OAS 1462	mat3-Mc F	cgggttcccctatttcctac
OAS 1462	mat3-Mc R	ttgaggtcttggcagttgtg
OAS 1569	MAT distal F	TTTGTTTGGCCGATAGTCTG
OAS 1570	MAT distal R	GTTATGTTGGCAAAACGATTGCT
OAS 1601	mtd1 F	CCTGCCAGCAAGGAGAGGTT
OAS 1602	mtd1 R	TGGTACTTACTGCAGCTATGGTTAGG
OAS 1822	mei4 F	CCCCCTCTTCCCCCTTCTAA
OAS 1823	mei4 R	AAAGGCTAGACTCATGGGCATC
OAS 2087	mcp7 F	GGAAGCTGCCAATTTGTGGA
OAS 2088	mcp7 R	TTGCAGATCGTCCAAATCCTC
OAS 2091	ssm4 F	CAATTCCAAGCCCATTGACC
OAS 2092	ssm4 R	TGAAAACTGGGTCGCTCGAT
OAS 2097	SB_TEL1L_3 F	TAATGAGTTGCCCCGGGTAT
OAS 2098	SB_TEL1L_3 R	CCGAATGGCAAGATGGTAAT
OAS 2099	SB_TEL1L_7 F	CACAGACGTCTCCTGGTGTC
OAS 2100	SB_TEL1L_7 R	TGCAGAGTTTGCGGTACTTG
	swi6	GGTCGTCGGACAATGAAGTT
	swi6	TCCACCTTTTCTCGCCATAC
	clr4	TTCAAAGAAGCTGGGGAAGA
	clr4	CGTTACTTCGGGTCCTTCAA
	rik1	CGGTAGACACAGCGACTTCA
	rik1	TAATCCTGGAGGGCGATATG
	clr6	ATTTCTGCTGGCGGTTCAAT
	clr6	TCTACACCGTCTCCGTGGTG
	prw1	AGTGCGAAGCTTATGCCACA
	prw1	TAGCGTCTTGCGATCCAGAA
	clr3	AGACGCGTCCTTCGTGTTTT
	clr3	CGAGCACAAAACGCACTTTC
	chp2	AAGAAGATTGTGCGGGCTGT
	chp2	GCTGCATTCCTGCTTCTCCT

sir2	GGGGCAGGCATAAGTACAAG
sir2	ATTCGTCTCCGGCAAAAGAT
chp1	AAATGGGCTGGCTATGATTG
chp1	TTGCGCTTTTGTCTTTGTTG
