# Supplementary material for: Local chromatin context regulates the genetic requirements of the heterochromatin spreading reaction
Source: PLoS Genet. 2022 May 18;18(5):e1010201. doi: 10.1371/journal.pgen.1010201 (PMC9154106; doi:10.1371/journal.pgen.1010201)
Supplement: Attachment — Submitted filename: response to reviewers, Greenstein Ng et al .pdf [file pgen.1010201.s019.pdf]

Response to reviewers:

We would like to thank the reviewers for their positive disposition and constructive comments. As major experimental additions in this revision, in response to reviewers #1&#2, we

1. performed H3K9me3 ChIP-qPCR at several loci (see new Supporting Figure 13)
2. introduced *clr6-1* and a *Δprw1* mutations in a reporter-less wild-type strain background and queried H3K9me2 by ChIP-qPCR at MAT (see new Supporting Figure 11)

Finally, we provide expanded analysis of our screen hits in response to reviewer #2. We also rewrote and edited the manuscript at key places to address the concerns of all reviewers (key passages highlighted).

Our point-by-point responses are below.

Reviewer #1: “Local chromatin context regulates the genetic requirements of the heterochromatin spreading reaction” by Greenstein et al.

Heterochromatin is a repressive chromatin structure that can spread across large domains to enforce stable silencing of gene-poor regions containing repetitive elements. Heterochromatin domains can also form across gene-rich regions to elicit the signature gene silencing pattern of a specific cell lineage. In this manuscript, the authors investigate how genomic context contributes to heterochromatin spreading activity by using a quantitative silencing assay in combination with a genetic screen, which allows them to uncover factors that impact heterochromatin-mediated silencing at locations distal to a nucleation site. The authors report that the factors that support spreading do indeed differ depending on genomic context. From their analyses, the authors conclude that the Clr6 deacetylase is recruited by the FKH2 transcription factor specifically at distal sites of constitutive heterochromatin formation, but not within euchromatin or near nucleation sites. By contrast, Clr3 deacetylase is more generally required for spreading from nucleation sites throughout the entire domain. The authors also report that chromatin remodelers serve to antagonize heterochromatin spreading at distal sites.

Critique: This is an interesting study. The authors have identified several factors that differentially affect the heterochromatic silencing of reporters in different chromosomal contexts. This is highly significant as the factors identified in their screen would serve as an important community resource. Although it is clear from their extensive analyses that Clr6 deacetylase complex specifically affects silencing of reporter inserted distal to the nucleation sites, the interpretation that the observed changes are due to defects in spreading of heterochromatin is not fully supported by the results (e.g. Supporting Fig 7 shows little or no change in H3K9me2 levels across the MAT). The text needs to be revised extensively to more accurately describe the results presented. Specifically, the suggestions throughout the manuscript (and in the title) that the observed effects indicate changes in heterochromatin spreading should be removed before submitting a revised version for publication in PLoS Genetics.

We thank the reviewer for this generally positive assessment. We have provided now additional support that defects in Clr6 components result in defective spreading of H3K9me3, the functional silencing mark. I.e., H3K9me3 is not altered within nucleation sites but is compromised in distal regions, similar to H3K9me2.

For the factors where we do document this decrease in H3K9me3 (chiefly Clr6), we are comfortable terming these effects spreading defects as per the most common definition (an inability to extend a heterochromatin domain beyond sites where DNA sequences directly or indirectly recruit heterochromatin machinery, see also our definition for ChIP-seq, lines 339-342). We have been careful in this manuscript to refer to the loss of "orange" silencing as loss of "nucleation-distal silencing" wherever we have not specifically investigated heterochromatin structural components, like H3K9 methylation. However, we generally feel that loss of heterochromatin spreading is the most parsimonious explanation for loss of silencing at distal but not proximal locations relative to a nucleation site. In our prior work, we have extensively investigated the relationship between our heterochromatin spreading reporters and both, H3K9 methylation and antagonizing marks, revealing a strong positive and negative relationship, respectively (Greenstein et al. 2018) This has also been borne out in the context of mutants (Greenstein et al. 2019). However, for all mutants where we do not specifically document how H3K9 methylation is affected, we have now stated in this version that it is possible that distal silencing is lost even in the context of unaltered heterochromatin structure (lines 181-182). We hope the reviewer finds this approach acceptable.

Specific comments:

(1) The authors suggest that Clr6-related mutants affect the spreading of heterochromatin to mediate silencing of nucleation distal reporter. Whereas it is clear that Clr6 mutants differentially affect the silencing of nucleation proximal and distal reporters, these mutants show no major defects in heterochromatin spreading (e.g. see supplementary Fig. 7B). An important possibility is that the observed changes in gene expression are due to local chromatin changes in mutant cells rather than due to broader defects in the spread of heterochromatin. This possibility is supported by changes in H3K9me2 levels specifically near the orange reporter that is derepressed in Clr6 mutants. Related to this point, it would be better if the authors mapped the distribution of H3K9me3 as the spread of heterochromatin in S. pombe has been shown to require binding of histone methyltransferase Clr4 to H3K9me3.

We thank the reviewer for raising this point. It is correct that in the intact MAT locus, Clr6 components in our ChIP Seq appear to have very localized effects, specifically to the right side of *cenH.* These effects, while local, are not dependent on the reporter gene: We now submit evidence using a reporter-free MAT locus (see Supporting Figure 11) that *clr6-1* and *Δprw1* cause a decrease in H3K9me2 at *cenH*-distal sites. In fact, in this experiment and similar to the ChIP-seq results, it appears that H3K9me2 is elevated at *cenH* but reduced at Mat3-Mc and a more distal site. This would be consistent with a model where H3K9me2 (but not me3) heterochromatin accumulates onto nucleation sites, as it is unable to extend outward. This is also consistent with the unperturbed heterochromatin assembly in these mutants. Together we believe this indicates a specific defect in extending the repressive domain. However, as we now state in the revised version, Clr6 may work redundantly with other, previously described, factors to effect heterochromatin domain extension in the MAT context.

Further, we want to point out that heterochromatin spreading is disrupted at multiple loci in Clr6 mutants: 1. the left and right side of pericentromeric heterochromatin (Figure 5D Supporting Figures 9 C&D). The range of this spreading in the wild-type is restricted by barrier tRNA genes and essential genes such as *rpb1* (*cen II* L) at relatively close distances (~ within 5kb) from the pericentromeric repeats. However, our data convincingly show that heterochromatin becomes restricted to the regions containing nucleating repeats in Clr6 mutants. 2. We observe even

stronger spreading defects at subtelomeres, which likely present the hallmark of long-range spreading in yeast (Figures 5E&F and Supporting Figure 9 E&F). 3. We observe a nearly complete loss of H3K9me at heterochromatin islands in clr6 mutants but remarkably not in a clr3 mutant (Figure 5 G-I). Hence, despite local effects at the MAT locus, we feel that Clr6 is broadly involved in regulating heterochromatin spreading in *S. pombe.*

Finally, we also addressed the reviewer's point on H3K9me3. We conducted H3K9me3 ChIP in our *ΔREIII* HSS strain in the wild-type, *Δfkh2,* or *clr6-1* strains. We find for H3K9me3 a similar behavior to H3K9me2, revealing a loss at distal but not at nucleation sites. We observe this for the MAT locus, the pericentromere (left of *cen II*), subtelomeres, and islands (see new Supporting Figure 13A-D). As observed in our prior work at MAT (Greenstein et al. 2018), H3K9me3 and H3K9me2 decrease in concert at distal locations. The reviewer emphasizes the requirement of H3K9me3 for the spreading reaction and indeed, a loss of H3K9me3, therefore, is consistent with the loss of spreading we observe in Clr6 mutants.

(2) Similarly, the authors suggest that the Clr6 related mutants affect heterochromatin spreading at centromeres. While some changes in H3K9me2 levels can be seen, it is not clear whether these changes can be characterized as spreading defects. On the other hand, changes in H3K9me2 are observed at telomeres in the Clr6 complex mutant. However, it is unclear if Clr6 is directly involved and that these changes are functionally linked to changes in gene silencing.

Our new data on H3K9me3 (see our response to point 1) also support the loss of spreading at these sites. As mentioned above, the distances over which spreading is disrupted at the pericentromeres is relatively short, given the constraints imposed in the wild-type on the spreading distance from the repeat regions by barrier tRNA and, in some cases, by proximal essential genes. As for the subtelomeres, the observation that defects in H3K9me2 and H3K9me3 abundance are amplified with further distance from the nucleating sequences is another indication of spreading defects in our view. (As a side note, the sequences for nucleating sites is not available in the genome build for subtelomeric in all cases (see *tel* IIL, Figure 5E)).

For *clr6-1* itself, we observe increased H3K9me2 levels at/near nucleation sites that rapidly drop below wild-type beyond nucleation sites, suggestive of a restriction of heterochromatin in this mutant to nucleation regions, which is again consistent with a role in domain extension.

(3) The authors conclude that another deacetylase Clr3 is more broadly required for H3K9me2 and gene silencing, including at nucleation sites. As mentioned in the discussion, this conclusion seems to be different from previous studies that appear to have not observed any reduction in H3K9me2 at centromeric repeats in clr3Δ cells. They note that variable results might be due to a specific mutant (Clr3-D232N) used in their study as compared to clr3Δ used in previous studies. Since all functional assays (such as results of the genetic screen described in Fig. 1) were performed using clr3Δ, it seems important that these results are correlated with changes in H3K9me2 in the same strain (clr3 Δ). Also, the Clr3-D232N cells appear to maintain significant levels of H3K9me at the nucleation sites (Fig. 5 and S7); suggesting that heterochromatin assembles at nucleation sites in clr3 mutant cells but fails to spread to distal sites. The text in the abstract and main text should be modified to more accurately describe these results.

We thank the reviewer for this thoughtful comment. We note that our results provide functional silencing studies with both *Δclr3* as well as *clr3-D232N* alleles. Data in Figure 1 is *Δclr3,* while Figure 5C is *clr3-D232N.* We observe a qualitatively similar loss of gene silencing (Figure 1E,G,I MIDDLE vs Figure 5C). Therefore, the deacetylation function of Clr3 is required for its silencing phenotype and is strikingly different from *clr6-1*. It is indeed the case that variable degrees of H3K9me2 loss are observed across different studies in the literature, as we note. Ultimately, we do not know what leads to these variable results, but since those are evident for the Δ*clr3* null allele*,* these inconsistent results are not attributable to the nature of the allele. Further, given the largely invariant results we obtain for silencing for the delete and catalytic null, we have no reason to believe our ChIP-Seq and ChIP-qPCR results are misreporting the effects of Clr3 loss of function.

However, we do agree with the reviewer that our results do not necessarily require a binary explanation (i.e., with Clr6 involved in spreading and Clr3 in nucleation and basic heterochromatin assembly). As suggested by the reviewer, we now discuss alternative models in the discussion (lines 449-450), whereas we removed mentioning Clr3 in the abstract.

(4) It is argued that the Clr6 complex is specifically involved in distal “heterochromatin spreading” but is not required at nucleation sites. Considering that HDACs have overlapping functions, a possibility cannot be ruled out Clr6 involvement at nucleation sites is masked by other HDACs. The text needs to be modified and arguments about Clr6 acting downstream of Clr3 shall be reconsidered.

We thank the reviewer for this comment. We now consider in the discussion (line 472-473) this alternative scenario the reviewer points out. In other places in the manuscript, we softened the conclusion.

(5) The authors suggest that Fkh2 promotes heterochromatin spreading by recruiting Clr6 to distal sites. Since Fkh2 is a DNA binding protein, it is unclear how this factor targets Clr6 to distal regions. Does Fkh2 bind to specific sequences near the distal site?

We detail at least two models (see discussion) that are consistent with Fkh2 acting via its DNA binding domain: binding that is more degenerate than the consensus motif, or chromatin architecture pathways. While Fkh2 is described as a DNA binding transcription factor that recognizes specific motifs (Garg et al. 2015; Alves-Rodrigues et al. 2016), Fkh2 appears to be found, in support of the first model, on many regions of the chromosome that lack these specific motifs. These regions include the left side of the pericentromere, the MAT locus, and several other locations (pombase genome browser Fkh2 track, based on Garg et al. 2015). On the other hand, Fkh2 has also been described to be a chromatin-organizing protein (Knott et al. 2012). For instance, Fkh2 promotes chromatin loops and organizes origins of replication. It is therefore tempting to speculate that these clustering activities may also contribute to heterochromatin spreading, which would be consistent with several models of sequestered niches for heterochromatin assembly (Holla et al. 2020). However, the precise mechanism by which Fkh2 targets Clr6 to distal regions is, we believe, beyond the scope of this current manuscript.

(6) Loss of H2AZ negatively affects silencing of the distal marker, suggesting that it is required for “heterochromatin spreading”. The authors wrote, “H2A.Z is known to antagonize heterochromatin spreading in budding yeast (36), indicating that the role of this histone in preventing gene silencing by chromatin regulation is conserved.” How is function conserved? In

contrast to H2AZ, the loss of SWR1 complex involved in loading H2AZ has an opposite effect. This shall be explained in the text.

We are glad the reviewer caught an inadvertent misstatement. Our results indicate that H2A.Z plays a positive role in promoting spreading. A recent paper from the Murakami group also finds that H2A.Z is required for heterochromatin integrity in *S. pombe* in certain contexts, validating our findings (Tsukii et al. 2022). While H2A.Z regulation of heterochromatin spreading is indeed present in these two evolutionary distant yeast species, we now point out that the mode of regulation in S. cerevisiae is opposite to the one in S. pombe by revising our former statement ("**a** role of this histone in …" instead of "**the** role in preventing gene silencing", line 213-214).

Reviewer #2: The primary goal of this manuscript is to identify regulatory factors that enhance or suppress heterochromatin spreading. The authors use a very elegant two-color fluorescence reporter system to uncouple nucleation from the heterochromatin spreading. The authors primarily focus on spreading at the MAT locus and at an ectopic site where a dh reporter fragment has been inserted. The manuscript provides a comprehensive accounting of non-essential proteins that enhance or suppress heterochromatin spreading. The authors performed a very elegant screen to figure out which chromatin regulators enhance or suppress heterochromatin spreading. I also liked the idea of drawing a contrast between clr6 and clr3- both being histone deacetylases but one affecting spreading and the other affecting nucleation. The data about Fkh2 being in complex with Clr6 is very clear and the data is internally consistent in identifying Fkh2 as a top hit in the genetic screen.

The quality of the data, the analysis frameworks and the very careful discussion of the results are strengths of this manuscript. Heterochromatin spreading is dynamic and I really like that the authors use a fluorescence-based approach with the sensitivity to parse out different populations of cells to investigate this process. In summary, this is a technically solid manuscript, and the screen is very well designed. However, I do have some major concerns that should be addressed. The first of my comments is a conceptual issue I have with how the screen is presented and the other issues relate to additional experiments that are needed to solidify author claims. I support publication of this manuscript provided the authors can be responsive to these concerns.

We thank the reviewer for this positive assessment. We provide responses below.

1) The authors present findings of different chromatin regulators (this is more of an issue in the loss of function factors) whose deletions lead to an increase in heterochromatin spread. Three genetic contexts in their screen involve the MAT locus. When eliminating different nucleation and/or spreading elements (such as cenH and REIII), the authors find different lists of chromatin regulators that affect spreading in their screen. It is very likely that these lists are different because each represents a sensitized background of some kind where nucleation is affected (I am aware the authors pre-selected 'green' cells but heterochromatin is dynamic and pre-selecting green cells does not mean that nucleation is not sensitized to stochastic loss). It is very important that this potential pitfall is laid out very clearly in the design of the screen. Their results imply there is a hierarchy of chromatin regulators that affect spreading- some in strong nucleation contexts and others in weak or impaired nucleation contexts (such as REIII and cenH deletion). But it is very important to present the discovery of the different chromatin regulators in

a unified manner as it concerns the MAT locus. In my view, the authors are only comparing two loci- MAT and ECT and all other strains are a subset of MAT.

We thank the reviewer for raising this point.

1. We assume the reviewer is concerned that while we have stringent gates for the selection of “green-OFF” gates, it remains possible that, in some contexts, there could be a transient loss of nucleation in individual cells for some but not other strains. The following argues against this possibility: First, in our heterochromatin spreading sensor, the fluorescent proteins are quite stable, i.e. relatively long-lived (Al-Sady et al. 2016). Therefore, a loss-of-nucleation event will persist as a green ON signal for a few cell divisions. However, in the cell population of mutants, we isolate as spreading hits (orange is ON), we do not witness elevated green levels (which we would expect even if there were transient green expression). Second, the population distribution of green in all the MAT strains is a uniform Gaussian around the OFF state, indicating that loss events are very rare. (For ECT, the green distribution is different, though, because the insertion site of green here is proximal but not within the nucleation site.) These tight Gaussian OFF distributions (Greenstein et al. 2018) do not hint at a tail of differential nucleation failure events. However, extremely transient nucleation losses that are not recorded as bursts of green expression could conceivably occur differentially between backgrounds. We now explicitly note this potential caveat when we describe the screen (see lines 124-130).
2. We understand that the reviewer is pointing out that we are comparing three different MAT contexts (2 singly, 1 dually-nucleated) with 1 singly-nucleated ECT context. We agree that it would be clearer to compare similar categories of spreading behaviors. In Figure 1 (loss of spreading), we now show three contexts where nucleation proceeds <u>only from one site</u>: Either *cenH, REIII* (within MAT), or *dh* (at ECT). For gain of spreading, we now also show only the singly nucleated contexts we could analyze (new Figure 2). We also continue to show the comparison of all contexts, including the dually nucleated *WT-MAT* context, in Supporting Figures 2&4.

2) An extension of the previous comment is that figures such as the upset plots in my opinion become complicated by including REIII and cenH deletion strains as genetic contexts that are separate from MAT. Furthermore, presenting hits of their screen in the REIII and cenH deletion backgrounds as part of the main figures makes the manuscript feel a bit more tedious. However, I want to be clear that what the authors choose to do about their figures has no consequence for my review. This comment is merely to be treated as one person’s opinion of an area where I think the manuscript can benefit in terms of clarity.

We thank the reviewer for this comment. We want to clarify that dissecting heterochromatin spreading from different types of nucleation elements is a central objective of this paper, hence we included *REIII* (RNAi-independent), *cenH*, and *dh* (RNAi-driven). We apologize if the data have been presented in a less comprehensible way. As stated above we have now reduced Figure 1 to the singly nucleated contexts (Figure 1D-I). Further, to broadly compare chromatin environments (MAT vs ECT), we now provide an upset plot that combines all the MAT singly-nucleated data with ECT for a more straightforward comparison, see Figure 1K.

3) In Figure 5D, the authors present H3K9me2 ChIP-seq data. The PCA analysis and ChIP-seq tracks of clr6-1 clearly suggest that the H3K9me2 patterns look different from that of wild-type cells. However, in many cases H3K9me2 levels in clr6-1 strains are higher than that of wild-type

strains. This is a mechanistic detail that is important to a key idea in the manuscript. The authors should address this concern by including ChIP-qPCR H3K9me3 data (ChIP-seq is also fine) to determine if changes in H3K9me3 could explain the spreading defects that are unique to the Clr6 hypomorph. Without the H3K9me3 data (regardless of the outcome itself) it is unclear what the molecular nature of the spreading defect actually is!

We thank the reviewer for raising this issue. Our new data for *clr6-1* implies that H3K9me2 is trapped and accumulates onto the nucleating and nucleation-proximal regions, whereas spreading beyond those regions is abrogated (see new ChIP qPCR below and ChIP-Seq in Figure 5 and Supporting Figure 9). However, we agree that it is useful to also examine H3K9me3. We performed ChIP in our *ΔREIII* HSS strain in the wild-type, *Δfkh2,* or *clr6-1* strains. We find a broadly similar behavior to H3K9me2, indicating a loss at distal, but not nucleation internal sites (see new Supporting Figure 13). We observe this for the MAT locus, the pericentromere (left of *cen II*), telomeres, and islands. As observed in our prior work at MAT (Greenstein et al 2018), H3K9me3 and H3K9me2 decrease in concert at distal locations. Unlike H3K9me2, H3K9me3 does not appear to be elevated beyond wild-type levels at nucleation sequences. Overall, these data address the molecular nature of the spreading defect, i.e. the inability to extend both structural (H3K9me2) and repressive (H3K9me3) H3K9 methylation beyond nucleators.

4) The authors use two fluorescent reporters to visualize changes in gene silencing. The decrease in H3K9me2 levels in clr6-1 strains at the site of the orange reporter insertion is concerning (Figure 5D and related interpretations in Figure 5). Since the manuscript focuses on how local chromatin context changes affect spreading, the authors must test how insertion of their reporter is affecting spreading especially in some of their mutant contexts (clr6-1 should be sufficient). My recommendation for an experiment would be an H3K9me2 ChIP-qPCR measurement in a wild-type and clr6-1 strain where the orange reporter has NOT been inserted. Directly comparing the two results will reveal the extent to which the reporter itself affects the observation. This does not detract from the strength of the manuscript but will provide a note of caution for other ways in which their results can be interpreted.

We thank the reviewer for raising this important point about the effect of the reporter gene insertion. In the intact MAT locus, *clr6-1, Δfkh2,* and *Δprw1* (mutants in Clr6 components) in our H3K9me2 ChIP-seq appear to have localized effects, specifically to the right side of *cenH,* while this complex has broader effects elsewhere, i.e. at the borders of the pericentromere, the subtelomeres, and islands (Figure 5J, SFig 9B vs Figure 5D-I, SFig 9C-F). To test whether the local effects we observe at MAT are dependent on the reporters, we performed an experiment along the lines suggested by the reviewer: We assessed the impact of *clr6-1* and *Δprw1* on H3K9me2 in an unperturbed h^- mating type (with *cenH* and *REIII* intact). We find *clr6-1* and *Δprw1* decrease H3K9me2 only at *cenH*-distal sites. In fact, for *clr6-1,* in this experiment H3K9me2 is elevated at *cenH* but depressed at Mat3M-Mc and a more distal site (see Supporting Figure 11), consistent with the H3K9me2 ChIP-seq.

These data would be consistent with a model where H3K9me2 (but not me3) heterochromatin is restricted and onto nucleation sites and thus accumulates to higher levels, as it is unable to extend outward. This is also consistent with the unperturbed heterochromatin assembly generally in these mutants and, we believe, highly suggestive of a specific defect in extending

the domain. We do not know why the effect of Clr6 is relatively localized to the right side of *cenH.* The most likely explanation is that spreading is executed by a number of factors in concert at MAT, while Clr6 is more important at other sites. In the end for our screen, it was certainly fortuitous that the right side of *cenH* is sensitive to mutants in Clr6.

5) Regarding Fkh2, its DNA binding properties and mechanism of regulating heterochromatin spreading (Figure 6): If the authors are invested in the claim that Fkh2 acts independently of its DNA binding domain then its important they either mutate or delete the DNA binding domain. In the absence of including such data, the authors need to modify their interpretations. While they have assayed a handful of heterochromatin proteins for their mRNA levels, it is impossible to rule out there may be other factors (expected or unexpected perhaps) that are regulated by Fkh2. Making the relevant Fkh2 mutants (either DBD mutant or a point mutant) is essential to support their claims made in Figure 6 and the discussion section.

We thank the reviewer for raising this point about Fkh2. We are open to Fkh2 acting in a DNA binding motif specific, or non-specific manner and are not invested in a claim that it acts independently of its DNA binding domain. The data in Figure 6 shows that in the absence of Fkh2, Clr6 recruitment to nucleation-distal sites is reduced, which could occur by several different mechanisms. In fact, we detail at least two models (see original discussion) that are consistent with Fkh2 acting via its DNA binding domain: binding that is more degenerate than the consensus motif, or regulation of chromatin architecture via canonical motif binding. In support of the first, while Fkh2 is described as a DNA binding transcription factor that recognizes specific motifs (Garg et al. 2015; Alves-Rodrigues et al. 2016) Fkh2 appears to be found on many regions of the chromosome, including at the left side of the pericentromere, the MAT locus and several other locations (pombase genome browser Fkh2 track, based on Garg et al. 2015). On the other hand, Fkh2 has also been described to be a chromatin organizing protein that can promote chromatin loops and organize and cluster origins of replication (Knott et al. 2012). In this model, it may use these clustering activities to promote H3K9me-spreading within chromatin regions, consistent with several models of sequestered niches for heterochromatin assembly (see references in the discussion). We further clarify these possibilities in the revised version (lines 486-493). Of course, a possibility also remains that Fkh2 executes its Clr6 recruitment activities in a manner independent of its described DNA binding domain. The precise mechanism by which Fkh2 targets Clr6 to distal regions is, we believe, beyond the scope of this current manuscript.

Reviewer #3: Silent chromatin structure, heterochromatin can spread from the nucleation site. Fission yeast provides a good model to analyze heterochromatin formation and many research revealed that several nucleation systems and many factors for nucleation has been identified. But the mechanism and the factors for spreading are not well understood. Greenstein et al. tried to clarify this issue using their single cell analysis system that enable to quantitative measurement of the nucleation and the spreading of the silent state. Screening of deletion library revealed that specific genes are required for the spreading and the same different genes promote spreading from different nucleation sites.

This manuscript provides comprehensive list of genes required for spreading of silent state and suggests context-dependent spreading mechanisms. Though the quality of the data is high, the results are rather descriptive and lacking the detail of the mechanisms except the analysis of Fkh2-containing Clr6 HDAC complex. However, I think that the information in this manuscript

beneficial for many researchers working in chromatin and/or epigenetic field and suitable for publishing in PLOS Genetics.

We thank Dr. Murakami for this positive assessment.

Minor points
Supporting Figure 1B: MATDK should be MATDcenH

Thank you for pointing this out, we have fixed this now.

Figure 4 E-G and Supporting Figure 7 B-D: Fission yeast centromeres basically have symmetric repeats structure, but in the figures, allows, which (I suppose) represent repeats, are not symmetric.

Thank you for this comment. These feature annotations, including position and directionality with respect to the reference genome, were derived directly from the GFF (general feature format) annotation file from pombase. We recognize that there may be other annotations corresponding to these repetitive regions, with *dg* and *dh* repeats annotated, for example, but we aimed to utilize one annotation reference source throughout this study for consistency.

References

Al-Sady B, Greenstein RA, El-Samad HJ, Braun S, Madhani HD. 2016. Sensitive and Quantitative Three-Color Protein Imaging in Fission Yeast Using Spectrally Diverse, Recoded Fluorescent Proteins with Experimentally-Characterized In Vivo Maturation Kinetics. *PloS one* **11**: e0159292.

Alves-Rodrigues I, Ferreira PG, Moldón A, Vivancos AP, Hidalgo E, Guigó R, Ayté J. 2016. Spatiotemporal Control of Forkhead Binding to DNA Regulates the Meiotic Gene Expression Program. *Cell reports* **14**: 885-895.

Garg A, Futcher B, Leatherwood J. 2015. A new transcription factor for mitosis: in Schizosaccharomyces pombe, the RFX transcription factor Sak1 works with forkhead factors to regulate mitotic expression. *Nucleic acids research* **43**: 6874-6888.

Greenstein RA, Barrales RR, Sanchez NA, Bisanz JE, Braun S, Al-Sady B. 2019. Set1/COMPASS repels heterochromatin invasion at euchromatic sites by disrupting Suv39/Clr4 activity and nucleosome stability. *Genes & Development* **34**: 99-117.

Greenstein RA, Jones SK, Spivey EC, Rybarski JR, Finkelstein IJ, Al-Sady B. 2018. Noncoding RNA-nucleated heterochromatin spreading is intrinsically labile and requires accessory elements for epigenetic stability. *eLife* **7**.

Holla S, Dhakshnamoorthy J, Folco HD, Balachandran V, Xiao H, Sun LL, Wheeler D, Zofall M, Grewal SIS. 2020. Positioning Heterochromatin at the Nuclear Periphery Suppresses Histone Turnover to Promote Epigenetic Inheritance. *Cell* **180**: 150-164.e115.

Knott SR, Peace JM, Ostrow AZ, Gan Y, Rex AE, Viggiani CJ, Tavaré S, Aparicio OM. 2012. Forkhead transcription factors establish origin timing and long-range clustering in S. cerevisiae. *Cell* **148**: 99-111.

Tsukii K, Takahata S, Murakami Y. 2022. Histone variant H2A.Z plays multiple roles in the maintenance of heterochromatin integrity. *Genes to cells : devoted to molecular & cellular mechanisms* **27**: 93-112.
